# Aspartate in the Brain: A Review

**DOI:** 10.1007/s11064-025-04454-3

**Published:** 2025-06-12

**Authors:** Caroline D. Rae, Benjamin D. Rowlands, Vladimir J. Balcar

**Affiliations:** 1 Neuroscience Research Australia, Barker St, Randwick, NSW 2031 Australia; 2 School of Psychology, The University of New South Wales, Kensington, NSW 2052 Australia; 3 School of Science, The University of Wollongong, Northfields Avenue, Wollongong, NSW 2522 Australia; 4 Neuroscience Theme, School of Medical Sciences, Faculty of Medicine and Health, The University of Sydney NSW, Sydney, NSW Australia; 5 Laboratory of Neurobiology and Pathological Physiology, Institute of Animal Physiology and Genetics, Academy of Sciences of the Czech Republic, Brno, Czech Republic

**Keywords:** Neurotransmitter, Energy metabolism, Malate aspartate shuttle, d-aspartate

## Abstract

l-Aspartate (aspartic acid; C_4_H_7_NO_4_; 2-aminobutanedoic acid) is a non-essential α-amino acid found ubiquitously throughout the body, including in the brain. Aspartate is one of the protein-forming amino acids and the formation of tRNA-aspartate complex is catalysed by aspartyl tRNA synthetase. Free aspartate, which is the main subject of this review, plays key roles in metabolism, as an amino donor and acceptor. It contributes to the synthesis of protein, arginine and nitric oxide, asparagine, *N*-acetylaspartate and *N*-methyl-d-aspartate. Its major metabolic role in the brain is recycling reducing equivalents (protons) between the cytoplasm and mitochondrial matrix as part of the malate-aspartate shuttle. l-Aspartate’s actions on synaptic receptors, as well as its possible presence in nerve terminals and synaptic vesicles, are, in principle, consistent with a role as an excitatory neurotransmitter. The evidence is far from conclusive and at times controversial. The role of d-aspartate in brain function is even less certain but, it appears that, rather than being a minor neurotransmitter, d-aspartate is more likely to be involved in fine regulation of endocrine and homeostatic processes. Much research remains to be done in this area. The diversity of its functions and chemistry make aspartate a complex molecule to investigate and measure in vivo. Perturbations of aspartate metabolism have been described in a range of neurological deficits, particularly those of white matter. Here, we examine what is known about the various roles of aspartate in brain, its metabolism, transport and compartmentation, its role as a neurotransmitter or a more general signalling molecule, and what is currently known about its role(s) in disease processes.

## Introduction

l-Aspartate (aspartic acid; C_4_H_7_NO_4_; 2-aminobutanedoic acid [1]) was first identified following the earlier discovery of asparagine [2] in experiments where asparagine was heated with lead hydride and a crystalline acid was obtained [3], but it was not until later that the correct formula for aspartic acid was identified [4]. Aspartic acid was later identified to be a decomposition product of animal proteins [5] and, as such, is found ubiquitously throughout the body.

## Aspartate in the Brain—Where is it found and How much is There?

Aspartate is found in all brain cells, although the concentrations reported vary with values ranging from ~ 0.2 to 4–5 mmol/L. Initial reports of aspartate levels in the brain were mostly recorded in cell cultures or extracted brain tissue. The study of Urenjak et al. [6] reported higher levels in cultured Wistar rat neurons (2.59 mmol/100 mg protein) and oligodendrocytes (3.6 mmol/100 mg protein) compared to astrocytes (0.35 mmol/100 mg protein). Measurement of aspartate in rat hippocampus by HPLC–UV determined a value of 1.59 ± 0.22 μmol/g tissue [7]. Cortical values reported in mouse brains by magnetic resonance spectroscopy have ranged from 1.8–2.3 μmol/g wet weight, in rat brains 1.9–2.6 μmol/g.

A post-mortem study measured brain levels of aspartate across 50 discrete brain areas in adult and old humans [8]. Values in adult brains ranged from a high of 3.28 (μmol/g fresh tissue) in the ventral thalamic nucleus to a low of 1.1 in the cerebellar nuclei, while the highest value in the brain from old people was 4.64 (μmol/g fresh tissue) in the tegmentum pontis with 0.7 (μmol/g fresh tissue) also in the deep cerebellar nuclei. The human pattern of concentration was reported to differ from that of rats [8]. While there was a positive relationship between the values of aspartate found in adult brains with aspartate values in older brains (Pearson r^2^ = 0.74, P < 0.0001) there was no significant relationship between brain aspartate and brain glutamate levels in adults (r^2^ = 0.03; P = 0.25) nor in old brains (r^2^ = 0.06; P = 0.07). A more recent study using a novel liquid chromatography/mass spectrometry approach reported human frontal lobe aspartate concentrations in controls (73 ± 7 y) of 0.245 ± 0.063 μg/mg tissue (equivalent to 1.84 μmol/g) [9]. Values measured in post-mortem brain and in extracts must be viewed with some scepticism due to the metabolic breakdown that occurs in hypoxic tissues unless immediately frozen or microwaved [10].

In humans (Table 1), aspartate concentrations have been measured across a range of field strengths using magnetic resonance spectroscopy in the occipital cortex and a limited number of other areas, at concentrations from 2.1–3.1 μmol/g [11] although other more recent reports at higher magnetic field strengths have put the value somewhat higher [12, 13]. The measurements in vivo are also higher than the value reported from post-mortem brain in the occipital cortex (1.29–1.85 μ mol/g wet tissue [8]. In addition to the post-mortem vs living brain differences, this may be due to the difficulties in measuring aspartate levels by MRS (see Sect. “Measurement of Aspartate in the Brain with Magnetic Resonance Spectroscopy”) with subsequently high estimation variances.

**Table 1:** Outcomes and commentary of studies measuring aspartate in human brain using magnetic resonance spectroscopy

Disease	Subjects	Method/voxel	Anatomy	Outcomes and *Comments*	Reference
A: HV (Healthy volunteer)	7 HV	7 T s-LASER, 7 volunteers each measured multiple times at 4 sites. TE 30 ms, VAPOR water suppression, TR = 8 s, NA = 64, PCC = 20mm^3^, CR = 18 mm^3^	Posterior cingulate cortex (PCC) and corona radiate (CR)	Multisite study with sequence designed to be identical, including RF pulses at all sites. Partial volume correction with assumed water concentrations, fitted with LCModel PCC [Asp] = 3.8 ± 0.6 mM (N = 54) CR [Asp] = 2.9 ± 0.6 mM (N = 53)	[16]
A: HV	6 HV,	Spectra measured in same volunteers at 3 T and 7 T, SPECIAL 2 × 2x2 cm, TE/TR = 6 ms/4 s, NA = 128	Occipital cortex	3 T [Asp] = 3.1 ± 0.3; 7 T [Asp] = 2.9 ± 0.5 mmol/kg	[17]
A: HV	9 HV,	3 T HERMES TR/TE 2000/150, NA = 384 VAPOR water suppression, 5 × 3x3 cm	Right centrum semiovale	[Asp] 0.88 ± 0.17 mM *Edited spectroscopy*	[18]
A: HV	10 HV	4 T and 7 T. STEAM with VAPOR NA = 160 4 T TR/TM/TE = 5 s/42 ms/4 ms 7 T = 5 s/32 ms/6 ms	Occipital lobe	4 T [Asp] = 2.44 ± 0.19 7 T [Asp] = 1.97 ± 0.27	[19]
A: HV	8 HV	7 T STEAM TR/TM/TE = 5 s/32 ms/6 ms NA = 160 OVS and VAPOR water suppression. VOI 2cm^3^	Occipital lobe	[Asp] = 2.0 ± 0.4 mM	[20]
A: HV	23 HV 23 ± 4 y	7 T LASER TR 4.5 s, OVS and VAPOR water suppression. VOI 2.7 cm^3^, 2 cm^3^, 1.5 × 4 × 1.5 cm, 2.5 cm^3^, respectively	Occipital lobe Motor cortex basal ganglia cerebellum	2.9 ± 0.8 μmol/g 2.6 ± 0.3 μmol/g 1.2 ± 0.5 μmol/g 1.1 ± 0.4 μmol/g	[21]
A: HV	5 HV 28.0 ± 2.7 y	7 T s-LASER TR/TE = 1000/6.5 ms, VAPOR water suppression, VOI 3cm^3^	Parietal-occipital lobe	[Asp] = 3.8 ± 1.3 mM *Spectra adjusted for individual-specific macromolecules. “Averaged” T1 used*	[12]
A: HV	8 HV 6 M, 2F 29 ± 4 y	9.4 T, MC-sLASER TR/TE = 6000/24 ms VOI 2 cm^3^	Occipital lobe	Median [Asp] = 3.38 (3.13–3.81; 25–75% quartile) Not adjusted for relaxation. *Authors cite values as mM and note difficulty in comparing with other studies due to different methods used*	[22]
A: HV	Multiple subjects (25–49) both genders, ~ 20–40 y	3 T PRESS T2 measurements VOI 25 × 20x20 mm TR 1600, 100 TE steps (30–228 ms)	Periventricular white matter, occipital cortex & pregenual anterior cingulate cortex	T2 estimated at: PVWM 148 ± 21 ms OCC 90 ± 27 ms pACC 111 ± 20 ms *Indicates that tissue composition has strong effect on Asp relaxation times*	[23]
A: HV	Multiple subjects (19–33) 21.2 ± 3.3 y	3 T MEGA-PRESS, ACC 50 × 25x25mm, FWM 50 × 19x27mm; Visual cortex 20 × 40x30 mm; TR 2 s, TE 90 ms	Anterior cingulate cortex, frontal white matter, visual cortex	ACC = 1.69 ± 0.18 FWM = 0.93 ± 0.14 Visual cortex = 1.33 ± 0.35 Arbitrary units	[24]
Type-1 diabetes mellitus	32 T1DM 13 HV	4 T STEAM, TR/TM/TE = 4.5 s/42 ms/54 ms. VOI 25 mm^3^	Mostly grey matter occipital lobe & mostly white matter parietal-occipital lobe	Mostly grey matter HV [Asp] 2.51 ± 0.25; T1-DM [Asp] = 2.37 ± 0.25 mM Mostly white matter HV [Asp] = 1.19 ± 0.39; T1DM [Asp] = 1.28 ± 0.46 mM. Used an assumed water concentration for grey or white matter; LCModel No change in [Asp] between controls and T1DM patients *but note large SDs*	[25]
Short-sleep insomnia	12 short-sleep insomnia vs 19 normal sleep insomnia	3 T Asymmetric PRESS TE1/TE2 25/85 ms. VOI 2 cm^3^	Left occipital cortex	Reduced levels of Aspartate, glutamine and creatine in insomnia of short sleep duration vs insomnia with normal sleep duration. *A-PRESS, an uncommon sequence, in this case optimized for Gln*	[26]
Epilepsy	Epilepsy	^1^H NMR spectra of biopsied brain from patients with mesial or neocortical epilepsy	Cortex and white matter	Mesial Epilepsy cortex 2.0 (0.2) N = 29 White matter 2.03 (0.5) N = 21 Neocortical Epilepsy cortex 1.7 (0.2) N = 7 White matter 1.8 (0.7) N = 7	[27]
Treatment-naïve paediatric obsessive compulsive disorder (OCD)	13 OCD 11 HV	1.5 T PRESS TE = 30 ms	Right caudate nucleus, occipital lobe, cingulate gyrus, thalamus	No differences from control asp levels but positive correlation in right caudate nucleus between obsession scores and Asp/H_2_O. Pearson R = 0.65, P = 0.016. *Relatively large coefficient of variation for Asp vs other metabolites (e.g. 47% in occipital lobe vs 6% for NAA) suggesting measurement not reliable. Authors do not give individual metabolite fit information*	[28]
Alzheimer’s disease (AD)	Post-mortem brain. 10 AD, 4 non-AD with dementia and 4 controls	High resolution NMR of PCA extracts	Superior temporal, occipital cortex	Aspartate elevated compared to controls in superior temporal lobe and compared to non-AD dementia in occipital cortex. Asp levels positively correlated to post-mortem interval	[29]
Huntington’s disease (HD)	10 Early HD (45.6 ± 12.7 y) 10 HV (38.9 ± 13.8 y)	3 T s-LASER TE/TR = 28/5000 ms	Visual cortex Striatum	Visual cortex HV 3.11 ± 0.54; HD 2.87 ± 0.23 Striatum HV 2.18 ± 0.41; HD 1.68 ± 0.52 Aspartate decreased in HD striatum, no change in visual cortex. *Authors consider Asp estimations unreliable due to fit uncertainty*	[30]
Nicotine addiction	21 Nicotine addicts 24 non-smokers 40–60 yo males	3 T MEGA-PRESS TE/TR = 90/2000 ms	Medial pre-frontal cortex	Aspartate elevated compared to non-smokers. Aspartate positively correlated with daily smoking amounts	[31]

There were also a limited number of studies (not cited) where aspartate was included by the authors in the fitted basis set but either not reported in the final results or not highlighted due to the large variance in the data. Values are as reported in mmol or “institutional units”

*TR* repetition time; *TE* echo time; *NA* number of acquisitions; *TM* mixing time; *PRESS*, Point-REsolved SpectroScopy; *STEAM* STimulated Echo, Acquisition Mode; *s-LASER* semi-Localised by Adiabatic SElective Refocussing; *MC* metabolite cycled; *PCA* perchloric acid; *NAA N*-acetylaspartate; *HERMES* Hadamard Encoding and Reconstruction of MEGA-Edited Spectroscopy; *OVS* outer volume suppression; *SPECIAL* SPin-ECho, full-Intensity Acquired Localised spectroscopy; *VAPOR* VAriable Power with Optimised Relaxation delays

In healthy human cerebrospinal fluid aspartate levels are comparatively low and the measurements are somewhat confounded by the method employed as sample treatment which can potentially result in the release of conjugated aspartate. Taken together, the results suggest that levels of CSF aspartate rise with age. Results in disease states, such as amyotrophic lateral sclerosis, are more controversial with some reporting large changes in aspartate, NAA and NAAG [14] in ALS vs controls with a range of neurological disorders, others reporting variation with disease stage [15].

## Synthesis of Aspartate

Aspartate is synthesized from the Krebs cycle intermediate oxaloacetate (oxobutanedioate) by the enzyme aspartate aminotransferase (l-Aspartate:2-oxoglutarate aminotransferase; EC 2.6.1.1, also known as glutamic oxaloacetic transaminase, GOT) which catalyses the reversible transfer of an α-amino group between aspartate and glutamate (1):

1 $${\text{Aspartate }}\left( {{\text{Asp}}} \right) + \alpha - {\text{ketoglutarate}} \leftrightarrow {\text{oxaloacetate}} + {\text{glutamate }}\left( {{\text{Glu}}} \right)$$

It relies on pyridoxal phosphate (Vitamin B6) as a cofactor and is present in two isoforms, mitochondrial and cytosolic [32]. These two forms are homodimers and show a close homology. Human *GOT1* (cytosolic form) is located on chromosome 10 (at the interface between q241-q251 [33, 34]; and *GOT2* (mitochondrial) on 16q21 [35, 36].

The mitochondrial form of the enzyme (GOT2) plays a key role in the malate aspartate shuttle (see 5.2) and a role in the tryptophan pathway through irreversible transamination of kynurenine to form kynurenic acid. Kynurenic acid is the only known endogenous antagonist of *N*-methyl-d-aspartate receptors, and an antagonist at α7-nicotinic acetylcholine receptors. GOT2 is equivalent to kynurenine aminotransferase IV (KAT IV; [37, 38]).

GOT1 is highly conserved across pro- and eukaryotes. A rare heterozygous mutation has been reported in an Amish population, resulting in loss of serum GOT1 activity (~ 50%) with no reported metabolic abnormalities apart from a trend to higher fasting blood glucose. No homozygous mutants were detected [39]. A heterozygous mutation in GOT1 has been reported where a non-conserved glutamate is substituted for glutamine (p.Gln208Glu, rs374966349) which may cause familial macro-aspartate aminotransferase, a rare, benign condition [40]. In this case, high levels of serum GOT1 are reported in otherwise asymptomatic people, due to increased clumping of GOT1 with immunoglobulins in the circulation. There is no reported brain involvement or complications.

A heterozygous variant in GOT2 has recently been reported in a six-year-old boy with acquired microcephaly, severe seizure disorder, spasticity, sleep disturbances, abdominal spasms, and low levels of serine in plasma and cerebrospinal fluid with some symptoms responding to oral serine and pyridoxine supplements [41]. Some biallelic GOT2 mutations have responded well to similar supplementation [42].

## Degradation of Aspartate

Aspartate can be degraded by conversion to oxaloacetate, a catalytic intermediate of the Krebs cycle that is resynthesized in each turn of the cycle. The carbon backbone can leave the Krebs cycle as malate which can be converted to pyruvate by malic enzyme (malate dehydrogenase oxaloacetate decarboxylating EC 1.1.1.40) in a reaction requiring a cofactor NAD/NADP. There are three known isoforms; one cytosolic (*ME1*, Chr 6q14.2; [43]) and two mitochondrial forms (*ME2*, NADP^+^ requiring; Chr 18q21.2, [44] and *ME3*, (NAD^+^ requiring; Chr 11q14.2; [45]). The isoforms have quite different metabolic roles [46–48]. The different properties suggest that the cytosolic enzyme, which is both anaplerotic and cataplerotic, plays roles in supplying dicarboxylic acids for neurotransmitter formation, while the mitochondrial enzyme is mostly cataplerotic under normal metabolic circumstances, and is involved in the disposition of Krebs cycle intermediates [49]. Recent findings suggest that ME2 is crucial for mitochondrial pyruvate and energy metabolism, as well as cellular respiration [50] although its role in the brain needs further exploration.

Malic enzyme isoforms have been detected in neurons, with higher activity shown in rats by the mitochondrial form of the enzyme [51]. In rat astrocytes, it has been suggested that the reverse is true [47] with 95% of the malic enzyme activity in astrocyte-rich cultures attributed to the cytosolic form [48]. Activity of the enzyme(s) has been reported to be higher in cultured rat cerebellar neurons than in astrocytes with the activity in neurons in the carboxylating direction 2.5 times higher than the decarboxylating [52]. The fact remains that the ultrastructural determination of the location of these enzymes in the brain remains to be better determined as the role they play in metabolic compartments and in metabolism is still uncertain.

A locus containing *ME2* has been identified as common to the adolescent-onset idiopathic generalised epilepsy syndromes: juvenile myoclonic epilepsy, juvenile absence epilepsy, and epilepsy with generalized tonic–clonic seizures, increasing the odds of disease by six-fold [53]. The locus has also been associated with susceptibility to mania and psychosis [54].

## Metabolism of Aspartate

### Exogenous Aspartate

As an excitotoxic amino acid, exogenous aspartate has been shown to increase energy metabolism. A study in guinea pig brain cortical tissue slices showed increased metabolite pools and increased flux of ^13^C from [1-^13^C]D-glucose and [1,2-^13^C]acetate into downstream metabolites with stimulatory effects on both glycolysis and the Krebs cycle in the presence of 20 and 100 µM aspartate [55]. The lower concentration of aspartate had relatively strong effects on glial metabolism as measured by the incorporation of label from [1,2-^13^C]acetate, while labelling of citrate from both carbon sources was greatly increased at the higher concentration of aspartate [55].

### Malate Aspartate Shuttle—Linkage to Phosphorylation State

**Fig. 1:**
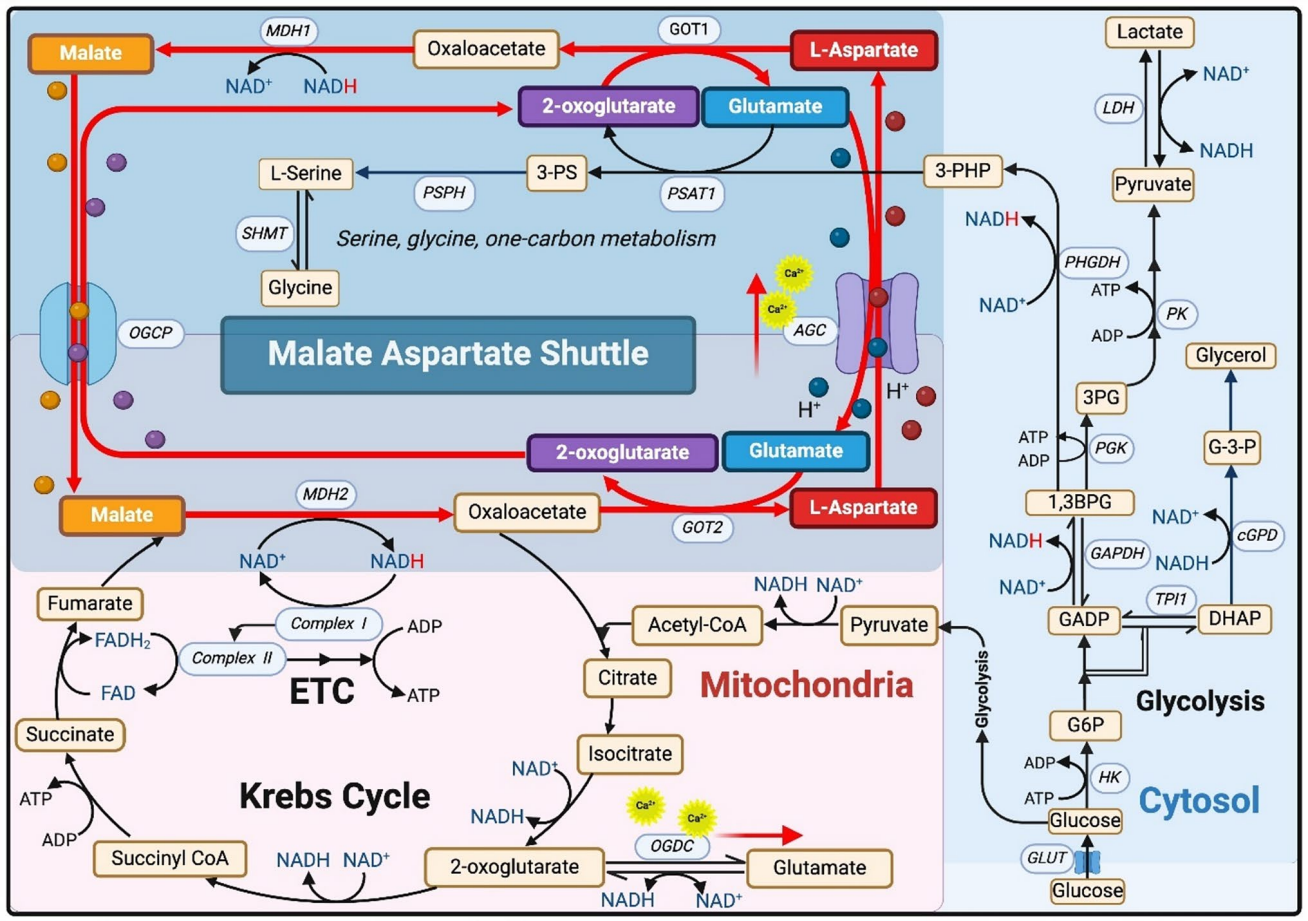
Scheme showing the malate aspartate shuttle. A proton (followed in red) produced by oxidation of glucose in the glycolysis pathway is transferred to the cofactor NAD^+^ forming NADH. This proton is transferred to malate via cytosolic malate dehydrogenase (MDH1; EC1.1.1.37). The malate is then transported into the mitochondrion in exchange for 2-oxoglutarate via the oxoglutarate carrier protein (OGCP) where it is converted to oxaloacetate by mitochondrial malate dehydrogenase (MDH2; EC1.1.1.37) and the proton transferred to NAD^+^ once more. By this mechanism, the cytosolic proton produced by oxidation of glucose can be transferred into the mitochondrion for subsequent introduction into the mitochondrial electron transport chain (ETC). The malate aspartate shuttle is then completed by the transamination, by mitochondrial aspartate transaminase (GOT2; EC 2.6.1.1), of the oxaloacetate to aspartate which is then electrogenically exchanged for glutamate via the aspartate-glutamate carrier (AGC). In the cytosol, the aspartate is converted back to oxaloacetate by cytosolic aspartate aminotransferase (GOT1; EC 2.6.1.1) forming oxaloacetate. This can then be converted to malate, picking up another proton from glycolysis in the process and starting the shuttle all over again. AGC contains calcium-binding domains and is stimulated by mitochondrial Ca2^+^, with activation enhancing the transport of aspartate and glutamate. Ca2^+^ activation of the 2-oxoglutarate dehydrogenase complex (OGDC; EC 1.2.4.2) drives the reaction towards glutamate, lowering the local concentration of 2-oxoglutarate which supports the transamination of oxaloacetate to l-Aspartate. Serine biosynthesis is indirectly influenced by the MAS as phosphoglycerate dehydrogenase (PHGDH; EC 1.1.1.95) activity is dependent on the NAD +/NADH ratio. When MAS activity is low, alternative NAD^+^ regeneration pathways, including lactate dehydrogenase (LDH; EC 1.1.1.27) and cytosolic glycerol-3-phosphate dehydrogenase (cGPD; EC 1.1.1.8), may partially compensate to support PHGDH function and sustain serine biosynthesis. Aspartate (red circle), glutamate (blue circle), 2-oxoglutarate (purple circle), and malate (orange circle)

The malate-aspartate shuttle (Fig. 1) plays a major role in the movement of reducing equivalents (NADH) into, or out of, the mitochondrion.

This is particularly important when the rate of glycolysis is high; production of NADH from glyceraldehyde-3-phosphate dehydrogenase activity decreases the cytosolic NAD^+^/NADH ratio. This NADH imbalance can be partially restored by other dehydrogenases such as lactate dehydrogenase and cytosolic glycerol-3-phosphate dehydrogenase (cGPD), but is mainly driven by the phosphorylation state of the cytosolic adenine nucleotide system (i.e. [ATP]/[ADP].[Pi] [56]). In the brain, as in the liver, the phosphorylation status determines the redox status, with the components of the aspartate aminotransferase maintaining equilibrium across a broad range of glycolytic activity [57]. Cytosolic aspartate aminotransferase (GOT1) converts cytosolic aspartate to oxaloacetate which can then be converted by cytosolic malate dehydrogenase to malate, regenerating cytosolic NAD^+^. The malate is then transported into the mitochondrion and reconverted to oxaloacetate by mitochondrial malate dehydrogenase, generating NADH within the mitochondrion; thus successfully transporting a proton from cytosol to the mitochondrial matrix, supporting oxidative phosphorylation.

Glycolysis is not the only generator of cytosolic NADH. The NAD^+^-linked phosphoglycerate dehydrogenase also generates cytosolic NADH when oxidising 3-phosphoglycerate, a key step in the serine and glycine biosynthesis pathway (Fig. 1). Defects in the malate-aspartate shuttle have been shown to disrupt NADH homeostasis in HEK293 cells, leading to lack of serine and impairments in one-carbon metabolism [58].

In glutamatergic neurons, glutamate-glutamine cycle activity is linked to the Krebs cycle via the “pseudo-malate-aspartate shuttle”. This posited that conversion of Gln to neurotransmitter Glu requires neuronal glycolysis, cytoplasmic NADH production, and redox and glutamate-carbon shuttling between cytosol and mitochondria and that this role was filled by the malate aspartate shuttle.This allowed the linking of neurotransmitter recycling with energy metabolism [59, 60]. The mechanistic details of how these two factors, which have 1:1 stoichiometry [61], are linked remain to be elucidated. A recently published paper proposed some improvements to the pseudo-malate aspartate shuttle model allowing it to comply with mass-balance and stoichiometric limitations (i.e. balancing products and reactants) [62].

### Malate Aspartate Shuttle—Components of the Shuttle

#### Aspartate Aminotransferase (GOT).

The two forms of GOT play a major role in the malate aspartate shuttle. Under activated conditions, the cytosolic form GOT 1 converts aspartate to oxaloacetate, providing a substrate for malate dehydrogenase and the regeneration of NAD^+^, while the mitochondrial GOT2 converts oxaloacetate to aspartate for export to the cytosol (Fig. 1). GOT has been shown to be a near equilibrium enzyme with slightly faster transaminase activity in synaptosomes than astrocytes [63], possibly due to the different, compartment-dependent, transient hetero-complexes it forms with other enzymes [64].

**Fig. 2:**
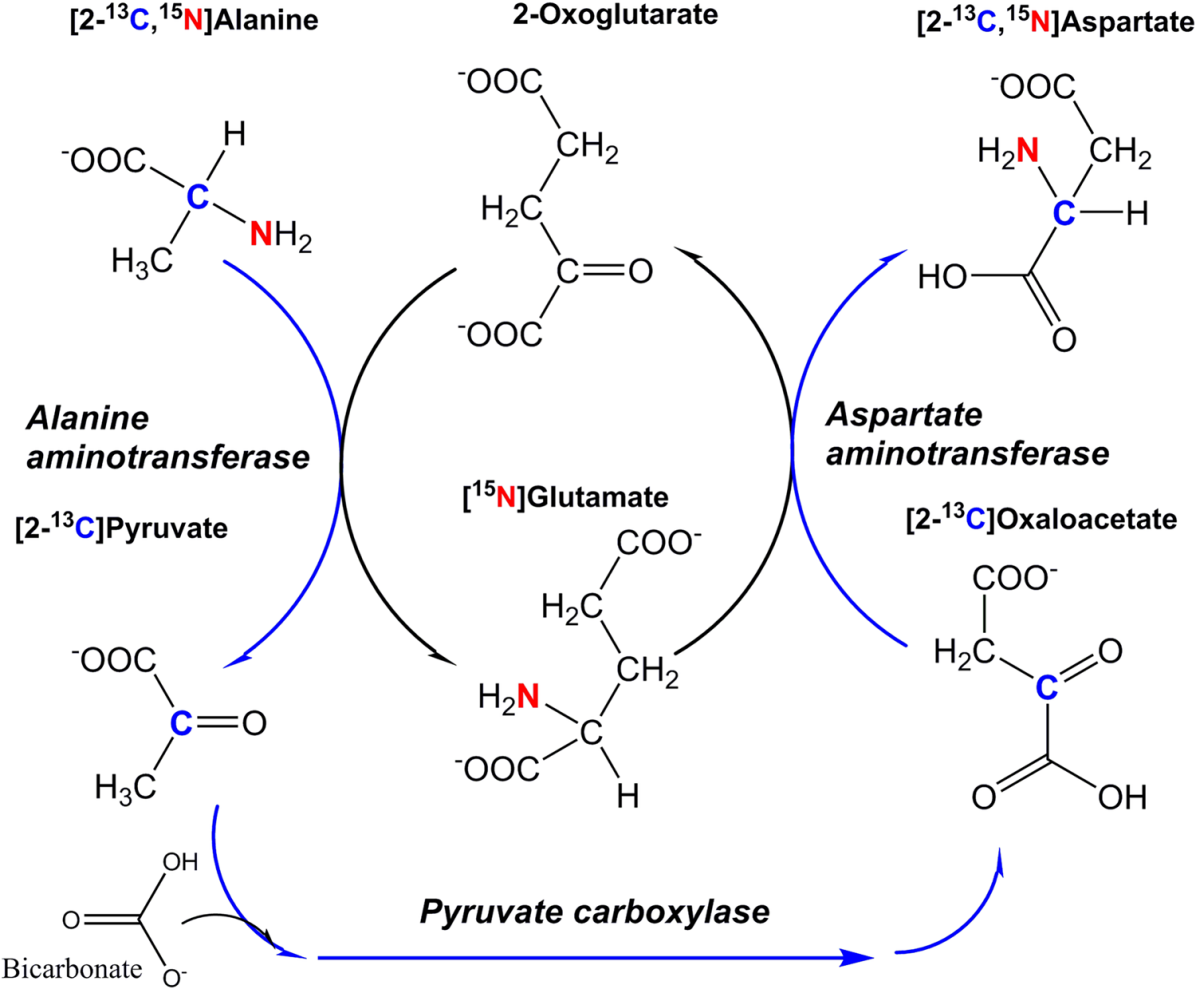
Scheme showing conversion of [2-^13^C,^15^N]alanine to [2-^13^C,^15^N]aspartate. The ^13^C is transferred by alanine aminotransferase from [2-^13^C,^15^N]alanine to [2-^13^C]pyruvate, which is converted to [2-^13^C]oxaloacetate by the glial enzyme pyruvate carboxylase. This carbon backbone is re-aminated by aspartate aminotransferase using [^15^N]glutamate to form [2-^13^C,^15^N]aspartate. Meanwhile, the ^15^N from [2-^13^C,^15^N]alanine is transferred to [^15^N]glutamate by alanine aminotransferase, thence to [2-^13^C]oxaloacetate by aspartate aminotransferase. The high level of ^15^N labelling in aspartate C2 indicated that these reactions were tightly coupled, and the need for pyruvate carboxylase showed that this tight coupling took place in glial cells. Scheme adapted from [65]

GOT is tightly compartmentalized with alanine aminotransferase (AAT; EC 2.6.1.2) in glial cells; a study using [2-^13^C,^15^N]alanine as substrate in brain cortical tissue slices produced almost exclusively [2-^13^C,^15^N]aspartate (Fig. 2). This could only happen if the carbon backbone from alanine was.

transferred to pyruvate via alanine aminotransferase, thence to oxaloacetate via pyruvate carboxylase and then to aspartate via aspartate aminotransferase all in the same compartment [65, 66]. Since pyruvate carboxylase is only found in glial cells ([67] although there have been recent reports that it is found in a subset of human neurons [68]), this indicated that the compartment in which this exchange was taking place must also be glial.

Under conditions of hypoglycemia aspartate is known to be elevated [69] as oxaloacetate accumulates in the Krebs cycle due to lack of available acetyl-CoA for citrate synthesis. Via equilibration through GOT this oxaloacetate is converted to aspartate, much of which is exported from the cell [65, 69, 70] although the transporter responsible for this export has not yet been identified.

#### Malate Dehydrogenase

(MDH; EC 1.1.1.37) has cytosolic (*MDH1*, chromosome 2p15, [71]) and mitochondrial (*MDH2*, 7q11.23, [72] forms which are ubiquitously expressed in the brain, rising to very high levels compared to the rest of the body as the brain develops [73]. Mitochondrial MDH (MDH2) is a highly active enzyme in brain [74] and the only enzyme in the Krebs cycle catalyzing a reaction with a highly unfavourable equilibrium (steady state equilibrium K_obs_ = 2.86 ± 0.12 × 10^–5^ [75]). It is allosterically regulated by citrate, with the production of oxaloacetate inhibited by high citrate and encouraged by low levels of citrate [76].

In the liver, MDH has been shown to operate well below its maximal velocity and well below the K_M_ values for NADH and oxaloacetate [77]. The rate of MDH in the brain has been estimated at 9 ± 2 μmol/g wet weight/min in anaesthetized adult rats [78].

It is likely that MDH2 is part of a complex of Krebs cycle enzymes [79]. In pig heart muscle an enzyme complex forms in mitochondria between GOT2 and MDH2 [80]. Formation of this complex is enhanced by the acetylation of lysine residues on GOT2 and the acetylated complex enhances the ability of the mitochondrion to produce ATP and increases mitochondrial NADH, NADPH and glutathione levels, while reducing production of reactive oxygen species (ROS) [81]; this work was done in pancreatic cancer cells and is controversial [82]. There is also evidence that the complex further associates with the inner mitochondrial membrane and 2-oxoglutarate dehydrogenase [83]. The lysine groups on GOT2 may be deacetylated by the mitochondrial silent information regulator SIRT3, which then impairs the ability of GOT2 to associate with MDH2. This post-translational modification of GOT2 provides another avenue through which the activity of the malate aspartate shuttle can be regulated. Another enzyme complex involving MDH2 has been described with citrate synthase in vitro [84, 85] suggesting that some enzyme conglomeration of MDH2 may well also take place in the brain. This has yet to be demonstrated in the brain, and it is not clear if such substrate channelling provides any thermodynamic advantage [82].

#### Malate Aspartate Shuttle Mitochondrial Carriers

##### Aspartate Glutamate Exchange

Mitochondria possess specialized carriers that play major roles in the malate-aspartate shuttle (Fig. 1). Aspartate-glutamate exchange is accomplished by carrier proteins. In mammals, there are two. AGC1 (Aralar, SLC25A12) and AGC2 (Citrin, SLC25A13) are members of a calcium-binding carrier subfamily with a bipartite structure; their C-terminals have features of the mitochondrial carrier superfamily while the N-terminals have EF-hand Ca^2+^ binding motifs. An EF-hand is a motif comprising two helixes joined by a loop of ~ 12 amino acids that can bind calcium; this motif appears throughout evolution in proteins such as calmodulin, troponin-C and many other Ca^2+−^sensing or Ca^2+−^dependent proteins. AGC1 (*SLC25A12*) and 2 (*SLC25A13*) are nuclear gene-encoded on Chromosomes 2 [86] and 7 [87], respectively. The protein catalyses the irreversible 1:1 exchange of aspartate for glutamate, importing glutamate and a proton, and exporting aspartate [88–91].

AGC1 and 2 differ both in tissue distribution [92] and calcium sensitivity [93] with AGC2 showing higher transport rates than AGC1 [91]. AGC1 is expressed strongly in the brain [92] and is the only isoform present in most neurons and neural stem cells, with expression levels increasing strongly during development [94]. It has also been reported to be expressed in glia Fluorescence-activated cells sorted from 10–12 week-old mice [95] and expressed strongly in adult rat brain fluorescence-activated cell sorted astrocytes [96]. Results using immunohistochemistry and in-situ hybridsation differ from those with fluorescence-activated cell sorting leading to some controversy about the expression of AGC1 in glia [97, 98]. AGC2 expression in adult mouse brains, is confined to discrete neuronal clusters [99]. AGC2 has been reported to be expressed in cultured glia [94] and in adult spinal cord but not in adult rat brain [94]. Expression of AGC2 is higher in deep cerebellar nuclei [99] suggesting that the cerebellum may have some reserve shuttle capacity in conditions of AGC1 deletion [100].}. Taken together, these results suggest that expression of AGC2 is likely confined to small populations of neurons in the adult brain.

Defects in AGC1 have been reported in humans [100, 101] with sufferers presenting with global developmental delay, intractable epilepsy (both focal and generalized), hypotonia and cerebral hypomyelination. Knockout mice have been generated that are born normally but display delayed development, dying at around three weeks of age. The brains are smaller with decreased amounts of myelin basic protein and decreased *N*-acetylaspartate (NAA) levels [102] which may derive from the large(80–90%) drop in aspartate levels seen in AGC1 deficiency [103]. Neuro2A cells, a proliferating neuroblastoma mouse line, generated with downregulated AGC1 display showed deficits in Complex 1 activity with increased production of reactive oxygen species (ROS) as well as reduced proliferation, which could be rescued when supplied with glutamine. These cells also showed reduced levels of NAA [104]. That AGC1 insufficiency reduced respiration in neurons but not in astrocytes has been confirmed by others, who suggest that astrocytic resilience may be due to the use of other NADH shuttles, such as the glycerol-phosphate shuttle [105]. Astrocytes also have other anaplerotic resources such as pyruvate carboxylase [106].

AGC1 deficiency has been shown to affect the proliferation of different brain precursor cells, including oligodendrocytes, where it has been shown to cause spontaneous and precocious differentiation of oligodendrocyte precursor cells into oligodendrocytes [107] and disrupted fatty acid synthesis [108]. This suggests a different route for AGC1 impairment of myelination beyond reduction in NAA (which is neither sufficient or needed to impact myelin production [109]) possibly via trophic factors [107].

**Fig. 3:**
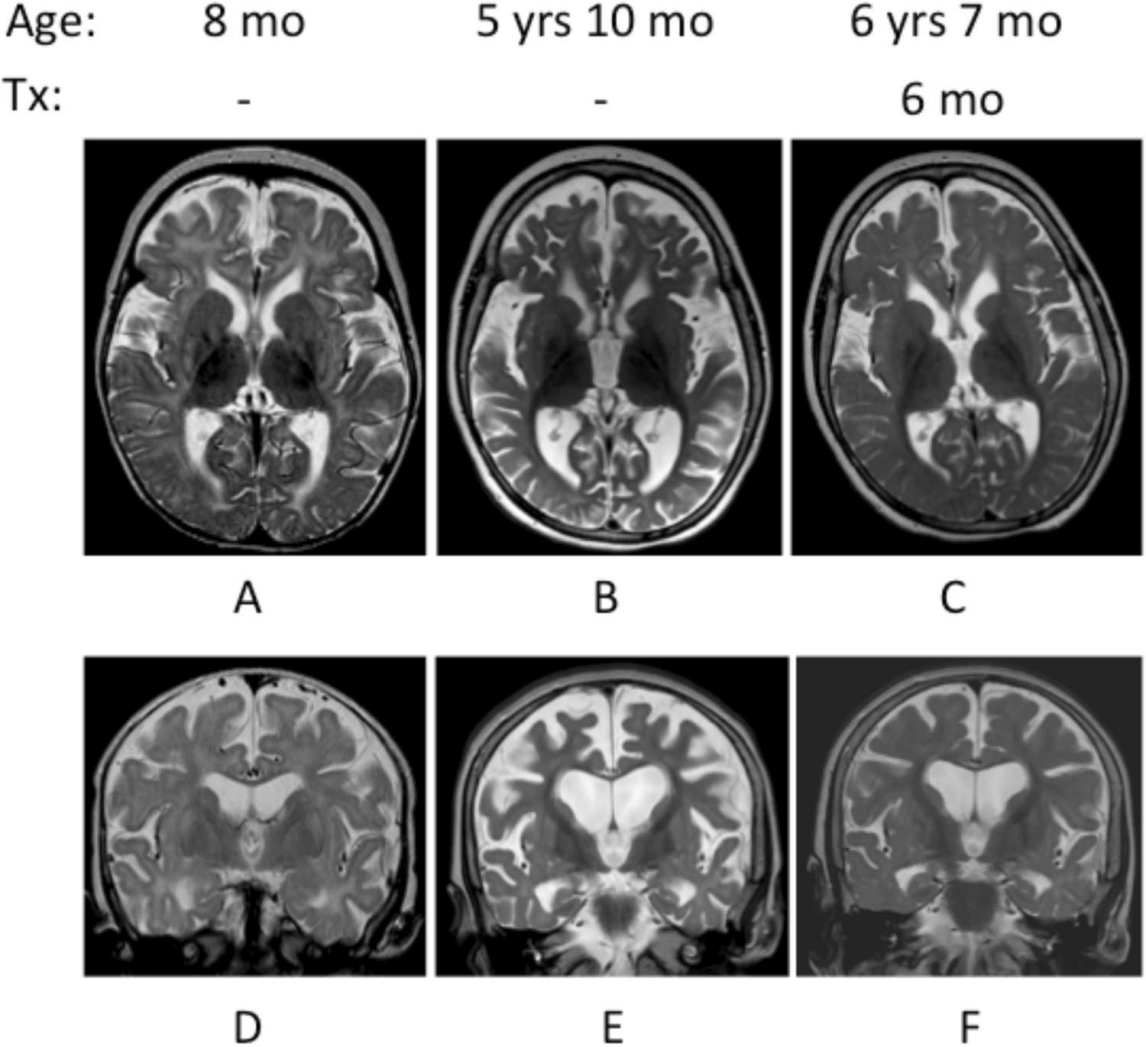
Results from MRI investigations in a patient with AGC1 deficiency before and 6 months after treatment [110]. Before treatment, T_2_ axial imaging **A**, **B** and T_2_ coronal imaging **D**, **E** showed lack of myelination and progressively reduced supratentorial cerebral volume. At 6 years and 7 months of age, after 6 months of treatment with the ketogenic diet, T_2_ axial imaging (**C**) and T_2_ coronal imaging **F** show that the previously high signal corresponding to white matter is lower, and that the ventricles and subarachnoid spaces are less prominent, indicating reversal of the volume loss. Tx, duration of treatment with the ketogenic diet. Reproduced from [110] with permission

Recently, patients with AGC1 deficiency have been shown to be responsive to a ketogenic diet [110, 111] with dramatic improvements in psychomotor development, cessation of seizures and increased brain myelination, including increased brain *N*-acetylaspartate levels [110] (Fig. 3). It was posited that the outcome was due to altering cytosolic redox through decreasing the relative amount of glycolysis to oxidative phosphorylation, since pyruvate administration, which may alter cytosolic NAD^+^/NADH has been shown to improve myelination in AGC1 knock-out mouse cerebellar slice cultures [102] but equally the ketogenic diet may alter mitochondrial redox [112] and boost Complex 1 activity [113].

Less clear is whether polymorphisms in the AGC1 gene (*slc25a12*) are associated with autism as studies in this area have yielded inconsistent results (e.g. [114, 115]). A recent meta-analysis points to the requirement for further work, with a small effect size and different sensitivity analysis outcomes depending on whether a family-based or case-controlled approach was used [116].

*Oxoglutarate/Malate Exchange* The other mitochondrial carrier important in the malate-aspartate shuttle is the 2-oxoglutarate/malate carrier protein (OGCP; SLC25A11) which catalyzes the electroneutral import of malate in exchange for 2-oxoglutarate with a K_M_ for 2-oxoglutarate of 46 ± 0.2 μM (rat liver; [117]). Although this shuttle is not Ca^2+^ dependent like the AGC it is responsive to mitochondrial Ca^2+^ via the enzyme 2-oxoglutarate dehydrogenase as the two proteins share a common substrate. Calcium entering the mitochondria activates 2-oxoglutarate dehydrogenase, thus altering the availability of 2-oxoglutarate and reducing OGCP shuttle activity [118]; oxidative phosphorylation and the malate aspartate shuttle thus compete for substrates and their activity is linked via mitochondrial Ca^2+^ availability [119]. The malate aspartate shuttle plays a key role in the regulation of aerobic glycolysis, whereby the activated brain preferentially upregulates glucose use compared with oxygen consumption, despite adequate supplies of oxygen, leading to the production of lactate and subsequent efflux of lactate from brain via diverse pathways [120].

Analysis of protein expression in infarcted human brain after ischaemic stroke has shown concomitant down regulation of all mitochondrial proteins (SLC25A11, SLC25A12, GOT2 and MDH2) involved in the malate aspartate shuttle [121]. In yeast, overexpression of mitochondrial components of the shuttle has been shown to extend longevity, independent of calorie restriction but via a SIRT-dependent mechanism [122]. Activity of mitochondrial enzymes is upregulated in human skeletal muscle by exercise [123], reduced by sleep deprivation [124] and decreases in rat liver with age [125].

#### Malate Aspartate Shuttle—Activity of the Shuttle

**Fig. 4:**
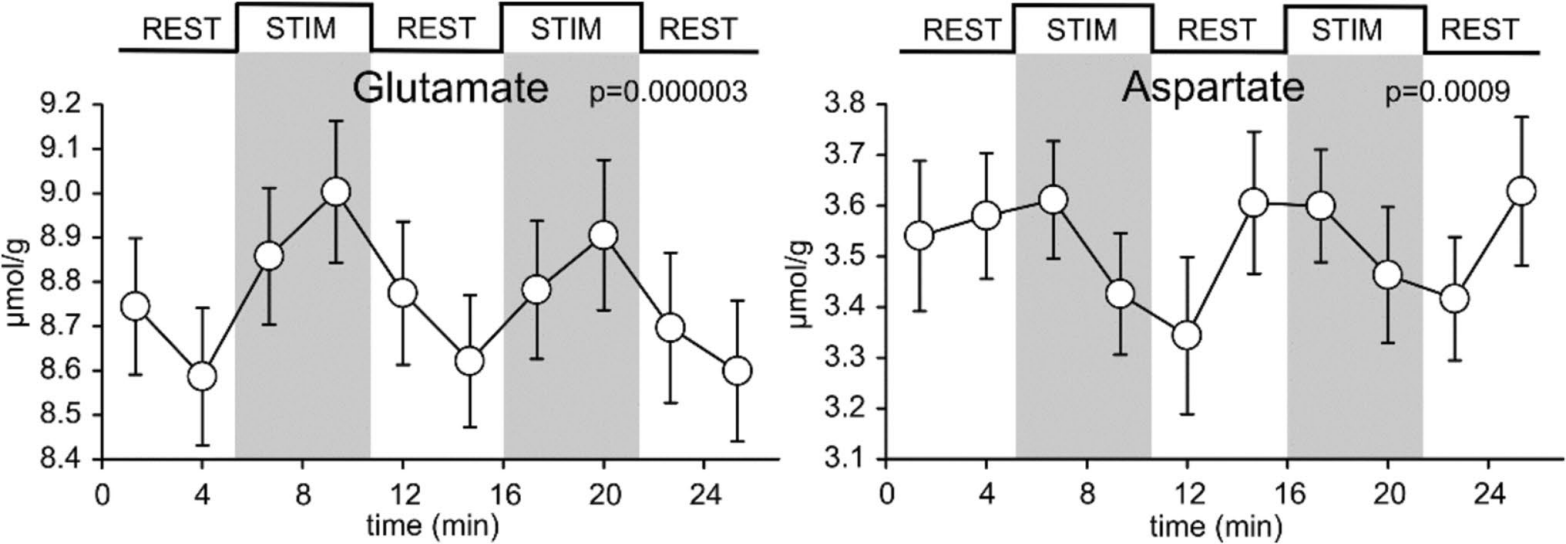
Time courses of glutamate (Glu) and aspartate (Asp) concentrations during a visual stimulation paradigm averaged across subjects (N = 12). Error bars indicate s.e.m., while shaded areas indicate the stimulation (STIM) periods. P-values correspond to statistical evaluation of differences between STIM and subsequent REST (resting) periods (paired t-tests, mean values from the second half of each period). Figure adapted from [13], with thanks to Silvia Mangia and Petr Bednařík (CMRR, U Minnesota)

The activity of the shuttle has been analysed in synaptosomes with the fastest reaction reported to be the one catalyzed by aspartate aminotransferase. The shuttle operates at a rate that seems faster than the Krebs cycle, but considerably slower than the rate of aspartate aminotransferase [126]. The rate of interconversion of 2-oxoglutarate and glutamate is similarly faster than the Krebs cycle, and has been estimated to be at least 80 fold faster [127]. Under normal conditions, the only irreversible step in the malate aspartate shuttle is the major regulating step: the activity of the AGC which has the lowest V_max_ of the shuttle components [119] and is driven by the proton electrochemical gradient and regulated by Ca^2+^ [128]. Where the activity of this transporter is decreased it would drive up cytosolic glutamate and decrease cytosolic aspartate levels (reviewed in [129]). This has been observed repeatedly (e.g. Figure 4) in conditions of neuronal activation in healthy control subjects [13, 130–132].

In practice, the malate aspartate shuttle is unidirectional toward oxidation of cytosolic NADH since efflux of aspartate from mitochondria is dependent on the proton-motive force generated by the respiratory chain; for every aspartate effluxed, mitochondria take up one glutamate and one proton [90, 133]. This explains why the mitochondrial NADH/NAD^+^ ratio is higher than in the cytosol [82].

Disorders of the malate-aspartate shuttle present with a clinical phenotype of infantile epileptic encephalopathy with biochemical characteristics that include high lactate, high glycerol 3‐phosphate, a disturbed redox balance, TCA abnormalities, high ammonia, and low serine [134]. The activity of the shuttle underpins important pathways such as glycolysis and serine biosynthesis. Inhibition of aspartate aminotransferase with β-methylene-D,l-Aspartate was shown to decrease oxidation of both glucose and pyruvate, decrease content of ATP and phosphocreatine as well as increase lactate/pyruvate (an indicator of cytosolic redox state) by three fold. The concentrations of malate, citrate and aspartate were decreased. Taken together, these results were held to show the importance of the aspartate aminotransferase in the malate aspartate shuttle and to show the importance of the ability of carbon to flux through the shuttle [135]. The interpretation of these results is complicated by the later finding that β-methylene-D,l-Aspartate inhibits glutamate uptake [136, 137].

In synaptosomes, inhibiting aspartate aminotransferase reactions with aminooxyacetate increases the mitochondrial NAD^+^/NADH ratio, while lowering ATP/ADP ratios and mitochondrial membrane potential [138]. The amount of extramitochondrial NADH oxidation is proportional to the number of components of the malate/aspartate shuttle that are introduced into the system [88]. Flux through the malate aspartate shuttle has also been shown to be essential for synthesis of neurotransmitter glutamate [60] as the aminotransferase route for the synthesis of glutamate from 2-oxoglutarate is more active than the direct amination (glutamate dehydrogenase) route [139]. In neurons, conversion of glutamine to glutamate during glutamate-glutamine neurotransmitter cycling was proposed by Hertz and Chen [140] to involve the malate-aspartate shuttle, and link cycling of one molecule of Gln/Glu to oxidation of 0.5 glucose molecules, accounting for half of the observed 1:1 stoichiometry of Glu-Gln cycling to neuronal glucose oxidation [141].

### Aspartate and Anaplerosis

The relationships between glutamine, glutamate and aspartate are also highly important. In developing brain it has been shown that the net flux of glutamine is from astrocytes to neurons where it contributes to build up of neuronal glutamate, aspartate and *N*-acetylaspartate pools [142], while neuronal aspartate is required by astrocytes for de novo synthesis of glutamate and glutamine [143]. Indeed the electron transport chain has been shown to be essential for the generation of aspartate in proliferating cells [144] with aspartate exported to the cytosol via the malate-aspartate shuttle for this purpose. The original suggestion that neuron-derived aspartate was required for glutamine synthesis [145] was based on studies in AGC1 deficient mice where the authors suggested that the carrier was only functional in neurons. This view was challenged by Hertz [146] who suggested an alternative model which was consistent with the observations of Pardo et al. [145] but allowed for generation of aspartate from glutamate in the astrocyte cytosol. Given that aspartate is an excitatory molecule (see below) it seems unlikely that aspartate would be transferred between cells for metabolic purposes. Hertz’s alternative model also satisfies stoichiometric considerations, which the Pardo proposal does not.

The relative amount of aspartate synthesized from glutamate appears to be species dependent, with mice using aspartate synthesis to cope with exogenous glutamate much more than humans, who tend to synthesise glutamine instead [147].

In a study of human cells with defects in the mitochondrial transport chain it has been shown that glutamine is essential for maintaining levels of aspartate; this flux can be stimulated with 2-oxoglutarate supplementation [148]. Similarly, inhibition of GABA breakdown with the GABA-transaminase (EC 2.6.1.19) and succinic aldehyde dehydrogenase (EC 1.2.1.16) inhibitor valproate has been shown to result in reduced aspartate concentrations that mirror the increases in GABA [149]. This anaplerotic activity producing aspartate may go some way to explaining the effect of mitochondrial inhibitors on levels of *N-*acetylaspartate [150] which would reduce concomitantly with aspartate levels.

### Aspartate and *N*-Acetylaspartate

Aspartate is a component of one of the highest concentrated amino acids in the brain, *N*-acetylaspartate (NAA), a compound that can be present in some neurons at up to 34.5 nmol/mg protein [151]. In healthy humans NAA levels in cerebrospinal fluid are negligible [152] with a mean of 0.51 (range 0.25–2.83 μmol/L) [153].

#### Synthesis of NAA

**Fig. 5:**
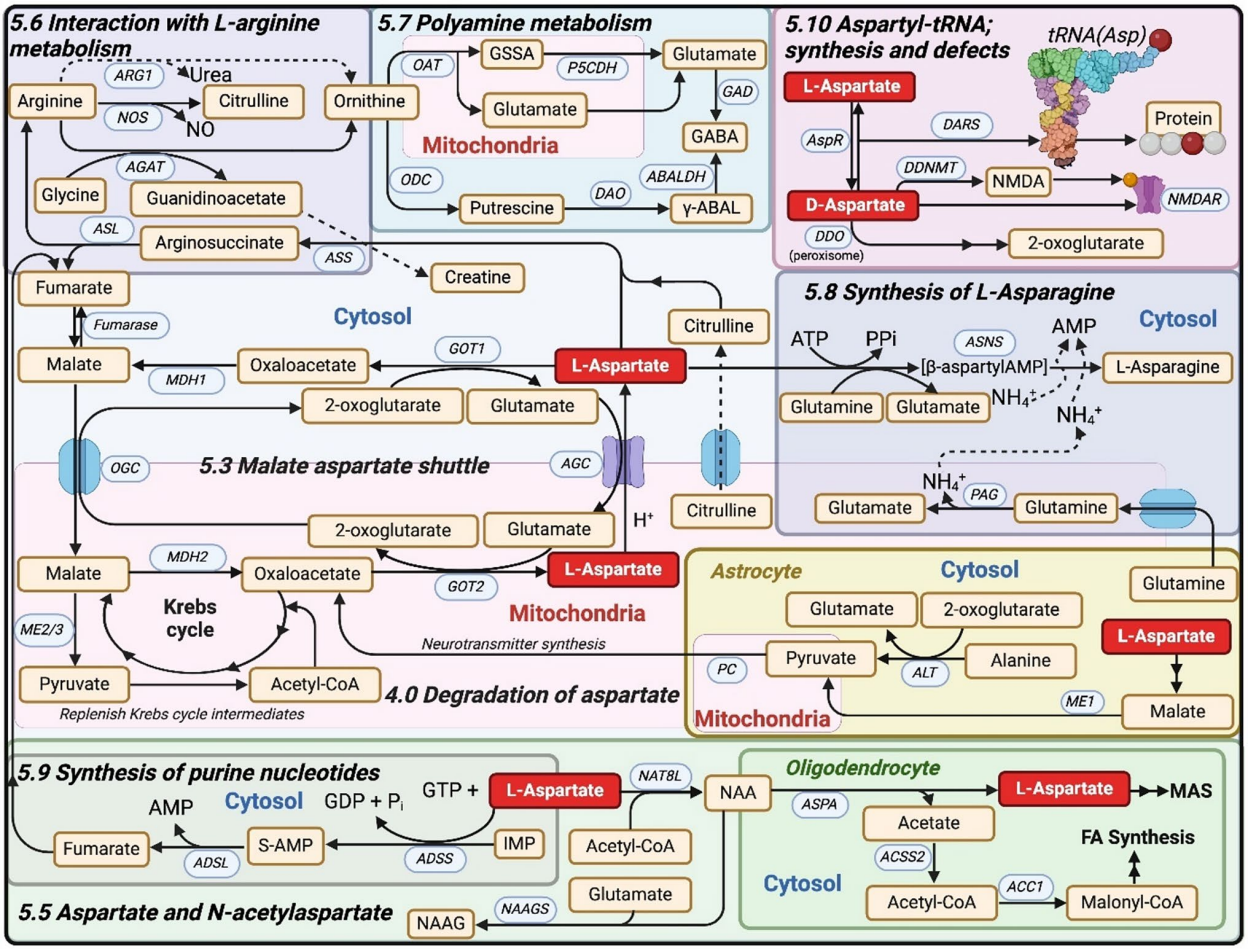
Metabolism of aspartate. Aspartate plays a central role in brain metabolism. This figure highlights several key metabolic pathways of aspartate metabolism that correspond to Sects. “Degradation of Aspartate” and “Metabolism of Aspartate” of the review. *5.3. Malate-Aspartate Shuttle*: Components of the shuttle are covered in Fig. 1. Key enzymes involved include alanine aminotransferase (ALT; EC 2.6.1.2), pyruvate carboxylase (PC; EC 6.41.1), aspartate aminotransferase or glutamate-oxalate transaminase 1 and 2 (GOT1/2; EC 2.6.1.1). malate dehydrogenase 1 and 2 (MDH1/2; EC 1.1.1.37), d-aspartate oxidase (DDO EC 1.4.3.1), aspartate-glutamate carrier (AGC), 2-oxoglutarate-malate carrier (OGC), fumarase (EC 4.2.1.2). *4.0 Degradation of aspartate:* Malic enzyme (ME1; EC 1.1.1.40) is both anaplerotic and cataplerotic, contributing to neurotransmitter formation via pyruvate carboxylase (PC; EC 6.4.1.1). ME2 (EC 1.1.1.38) and ME3 (EC 1.1.1.39) are primarily involved in mitochondrial pyruvate metabolism and Krebs cycle intermediate regulation. 5.5 *Aspartate and N-acetylaspartate:* NAA acts as an aspartate reservoir and is synthesised via acetylation of aspartate by N-Acetyltransferase-8 Like Protein (NAT8L; EC 2.3.1.17). In oligodendrocytes, NAA is hydrolysed by aspartoacylase (ASPA; EC 3.5.1.15) into aspartate and acetate. Aspartate may reenter the malate-aspartate shuttle (MAS), whereas acetyl-CoA synthesised from acetate by (ACSS2; EC 6.2.1.1) contributes to fatty acid (FA) synthesis via acetyl-CoA carboxylase 1 (ACC1; EC:6.4.1.2). NAA and glutamate may be metabolised into N-acetylaspartylglutamate (NAAG) by NAAG synthetase (NAAGS; EC 6.3.2.41). *5.6 Interaction with L-arginine metabolism:* Aspartate combines with citrulline via argininosuccinate synthetase (ASS; EC 6.3.4.5) to form argininosuccinate, which is cleaved by argininosuccinate lyase (ASL; EC 4.3.2.1) into fumarate that can be recycled in the malate aspartate shuttle, and arginine. Arginine is a metabolised by arginine:glycine aminotransferase (AGAT; EC 2.1.4.1) which converts glycine and arginine into ornithine and guanidinoacetate, with the latter being a precursor for creatine. Arginine can also be hydrolysed to ornithine by Arginase 1 and 2 (ARG1/2; EC 3.5.3.10) yielding urea. Arginine can be metabolised by nitric oxide synthase (NOS; EC 1.14.13.39) into citrulline and nitric oxide (NO). *5.7 Polyamine metabolism:* Ornithine is converted into putrescene via Ornithine Decarboxylase (ODC; EC 4.1.1.17). Putrescine is oxidized by diamine oxidase (DAO; EC 1.4.3.22) to form γ-aminobutyraldehyde (γ-ABAL), which is converted into γ-aminobutyric acid (GABA) by aminobutyraldehyde dehydrogenase (ABALDH; EC 1.2.1.19). Alternatively, ornithine and 2-oxoglutarate can be metabolised into glutamate-5-semialdehyde (GSSA) and glutamate by ornithine δ-aminotransferase (OAT, EC 2.6. 1.13). GSSA can be further metabolised by glutamate-5-semialdehyde dehydrogenase (P5CDH; EC 1.2.1.88) to glutamate. Glutamate can be converted into GABA by Glutamic acid decarboxylase (GAD; EC 4.1.1.15). *5.8 Synthesis of *l*-Asparagine*: Asparagine synthetase (ASNS; EC 6.3.5.4) hydrolyses glutamine, producing glutamate and releasing ammonia, ATP is then used to activate aspartate forming a β-aspartyl-AMP intermediate, which subsequently reacts with ammonia to generate asparagine and AMP. In brain, the source of this ammonia remains unclear. As phosphate activated glutaminase (PAG; EC 3.5.1.2) is localised within the mitochondria, any ammonia produced by this enzyme would need to be transported across the mitochondrial membrane. *5.9 Synthesis of purine nucleotides:* Adenylosuccinate synthetase (ADSS; EC 6.3.4.4) binds inosine monophosphate (IMP) and GTP is hydrolysed to GDP and Pi to condense aspartate with IMP to form adenylosuccinate (S-AMP). Adenylosuccinate lyase (ADSL; EC 4.3.2.2) then catalyses’ the cleavage of adenylosuccinate releasing AMP and fumarate. The fumarate can be recycled through the MAS and Krebs cycle, linking purine biosynthesis to energy metabolism. *5.10 Aspartyl-tRNA synthesis and defects:* Aspartyl-tRNA synthetases (DARS; EC 4.2.1.2) ensures proper aminoacylation of tRNA during protein synthesis (Asp moiety, red circle, other amino acids, grey circles). Aspartate racemase (AspR; EC 5.1.1.12) catalyses the conversion of l-Aspartate and d-aspartate. d-aspartate may act as an agonist at NMDA receptors, though its precise role in neurotransmission remains under investigation. d-Aspartate can be converted into N-methyl-d-aspartate (NMDA) by the enzyme d-aspartate Methyltransferase (DDNMT), which uses S-adenosyl-L-methionine (SAM) as a methyl donor. This conversion allows NMDA (orange circle) to act directly on NMDA receptors (NMDAR)

NAA is synthesized from aspartate and acetyl-CoA in a reaction catalyzed by *N*-acetyltransferase-8 like protein (NAT8L) and enzyme found in the mitochondria [154] and cytoplasm of neurons [155], neuronal microsomes [156] as well as possibly in oligodendrocytes during their development [157] (Fig. 5). One could view NAA as a significant sink for aspartate as the amount of aspartate confined in NAA far exceeds the concentration of free aspartate. Both lack of NAT8L and overexpression of it result in significant increases and decreases, respectively, in brain aspartate levels [109].

#### Degradation of NAA

NAA is hydrolyzed into aspartate and acetate by aspartoacylase (ASPA: EC 3.5.1.15) an enzyme solely located in oligodendrocytes [109, 158]. Aspartate generated from the catabolism of NAA may reenter the malate-aspartate shuttle (MAS), while the acetate moiety can be converted to acetyl-CoA via (ACSS2; EC 6.2.1.1) and may contribute to fatty acid (FA) synthesis via acetyl-CoA carboxylase 1 (ACC1; EC:6.4.1.2) (Fig. 5) [159]. Lack of ASPA causes Canavan disease [160, 161], a rare, autosomal recessive, neurodegenerative leukodystrophy associated with failure to reach developmental milestones, macrocephaly, seizures, oligodendrocyte death and widespread CNS vacuolization. This is associated with high levels of oligodendrocyte NAA. High levels of NAA in neurons caused by overexpression of Nat8L appear much more benign and do not cause leukodystrophy [109, 162] indicating that the presence of high levels of NAA in oligodendrocytes, or the inability to catabolize NAA in oligodendrocytes is the underlying cause of the leukodystrophy. High levels of NAA, which is a known osmolyte [163] have also been associated with increased levels of organic osmolytes in ASPA deficiency, such as taurine, *myo*-inositol and creatine [109].

Interestingly, high levels of NAA caused by a lack of ASPA do not result in decreased whole tissue levels of aspartate [109]. The aspartate derived from cleavage of NAA in oligodendrocytes is not exported and is thought to enter the malate aspartate shuttle and, eventually and slowly, be metabolized in the Krebs cycle [159].

NAA has been proposed as a protein stabilizer and aggregation suppressor, with the potential to solubilize preformed aggregates [164]. Other suggested roles for NAA include: establishing an improved diffusion microenvironment in some species in lieu of taurine [165]; mitigating proinflammatory responses in microglia [166] and driving oligodendroglial differentiation [167]via histone deacetylases.

#### Further Metabolism of NAA

NAA is also a substrate for N-acetylaspartylglutamate (NAAG). NAAG is the most abundant peptide in brain tissue and it has been argued that there is enough evidence to consider it a neurotransmitter [168] which would make this l-Aspartate containing molecule the second most abundant neurotransmitter, after l-glutamate, in brain [169]. High doses of NAAG administered directly into rat brain in vivo can be neurotoxic; perhaps even more neurotoxic than similar doses of glutamate given as a ‘positive control’ [170]. Whether NAAG is essential is questionable; a recently described mouse unable to synthesise NAA due to knockout of Nat8L has been shown to have no detectable brain NAAG while appearing generally normal [109]. Some minor slowing of the auditory startle response was reported but it was not possible to divorce the possible contributing effects of NAA, aspartate and NAAG in this case.

### Interaction with L-Arginine Metabolism

#### Interaction with Urea Cycle

Aspartate, as a substrate of the urea cycle, plays a role in the metabolism of arginine in brain [171]. Aspartate combines with citrulline in a cytoplasmic reaction catalyzed by argininosuccinate synthetase to form argininosuccinate (Fig. 5) which is subsequently cleaved to form arginine and fumarate by argininosuccinate lyase. Argininosuccinate synthetase has been shown to be localized to neurons in normal healthy brain, while expression of argininosuccinate lyase expression is found in both neurons and glia [172]. Under conditions of inflammation or hypoxia, expression of argininosuccinate synthetase is also found in glial cells, including astrocytes and microglia [173–176].

Aspartate is a precursor for the synthesis of arginine in the brain, providing an amine group for arginine synthesis. The backbone carbon subsequently produces fumarate and, via fumarase, malate, allowing the backbone the possibility of being recycled to aspartate via elements of the malate aspartate shuttle. Arginine synthesis has been reported to be a major fate for the amine group of aspartate [177] but the degree of urea production in brain is likely small. Arginase activity has been reported in brain including the liver style arginase 1[178] and the mitochondrial arginase II (low expression levels [179]) and production of urea reported in a number of brain preparations (e.g. [180]) but studies in astrocytes have reported minimal urea synthesis from aspartate [177]. In selected neurons, arginine is a precursor for the synthesis of nitric oxide [181, 182] and these cells express enzymes for the entire citrulline-NO cycle whereas numerous cells expressing arginosuccinate lyase do not express arginosuccinate synthetase, suggesting that exchange of intermediates between cells is a distinct possibility [172]. Arginine is a substrate for arginine:glycine aminotransferase (AGAT) which synthesizes guanidinoacetate, the precursor for creatine [183], such that aspartate provides a nitrogen group for the synthesis of creatine, a major energy currency in brain [184]. Aspartate is also used to make asparagine. The enzyme asparagine synthetase is cytosolic and it is described as using an ammonia group derived from glutamine. In brain, the source of this ammonia is not clear: phosphate activated glutaminase is a mitochondrially located enzyme [185] so if the ammonia is derived from this source it must exit the mitochondrion to do so.

#### Nitric Oxide

Arginine is the source of nitric oxide (NO; Fig. 5), originally discovered as a vascular relaxation agent but now understood to play multiple roles in the brain including regulation of the sleep–wake cycle, synaptic plasticity, hormone secretion and S-nitrosylation of a range of compounds [186, 187]. There are three subtypes of nitric oxide synthase (NOS); endothelial NOS (eNOS), mostly expressed in endothelial cells; neuronal NOS (nNOS) which is constitutively expressed in some CNS and peripheral neurons, and inducible NOS (iNOS) which is expressed in many cell types, including astrocytes and microglia, in response to cytokines and other inflammatory agents or challenges [188].

In general, cells capable of expressing nNOS also possess the full arginine recycling machinery, including both arginosuccinate synthetase and lyase [172]. Cells with iNOS upregulate expression of NO synthesizing enzymes following induced challenge but it is not clear whether this has an impact on aspartate concentrations.

Cultured astrocytes have been shown to readily convert ^15^N-aspartate into ^15^N-arginine [177]. This was the favoured pathway in cultured astrocytes incubated with 2.5 mM ^15^N-aspartate, incorporating more ^15^N than products of the aspartate aminotransferase reaction (^15^N glutamate, the ^15^N of which was subsequently transferred to glutamine, alanine, serine and ornithine) and more than production of pyridine dinucleotides (see below for more information on this pathway). When ^15^N-aspartate concentration was reduced to 0.5 mM, most of the nitrogen was recovered in alanine, glutamine and arginine and much less in [6-amino-^15^N]adenine nucleotides. This indicated that production of arginine is one of the major routes by which astrocytes dispose of excess aspartate [177].

### Polyamine Metabolism

The metabolism of ornithine, another urea cycle intermediate, is also of some importance for aspartate levels (Fig. 5). Ornithine can be converted to glutamate (and GABA) via ornithine decarboxylase (OCD, which converts ornithine to putrescine) or ornithine aminotransferase (OAT, which is the first step in a pathway which converts ornithine to glutamate and is probably of more importance in brain than the putrescine pathway [189–191]. Similarly to iNOS upregulation following an insult, ornithine decarboxylase is upregulated following a range of modes of injury to the brain, including chemical, physical and physiological (hypoxia) [192].

### Synthesis of l-Asparagine

l-Asparagine, the first amino acid to be isolated [193], is defined as a non-essential amino acid and is required for protein synthesis. Aspartate contributes to the synthesis in brain of l-Asparagine via a reaction catalyzed by asparagine synthetase (l-Aspartate:ammonia ligase (AMP forming); EC 6.3.5.4). The overall reaction (2) is as follows:

2 $${\text{L - Asp}} + {\text{ATP}} + {\text{L - Gln}} + {\text{H}}_{{\text{2}}} {\text{O}} \to {\text{L - Asparagine}} + {\text{AMP}} + {\text{PPi}} + {\text{L - Glu}}$$

l-Aspartate is activated by ATP and forms a β-aspartyl-AMP intermediate. l-glutamine provides an amino group that reacts with the β-aspartyl-AMP intermediate to form l-Asparagine, with glutamate, AMP and PPi being produced as well (Fig. 5).

#### Role of Asparagine Synthetase

Asparagine synthetase is primarily a cytosolic enzyme, with its activity in the brain about ten-fold less than in the pancreas but comparable to that in liver, kidney and spleen [194]. Although activity is relatively low in brain, the function it performs is important. While asparagine can be obtained via the diet, its concentration in plasma is low and its transport into brain relies on sodium-dependent amino acid exchange transporters, System N and System A [195]. These transporters do not actively accumulate amino acids and their kinetics in brain arguably act to *remove* asparagine from brain [196]. Consequently, deficiency in the activity of asparagine synthetase results in neurological symptoms, further underlining the importance of the activity of this enzyme.

Deficiency in asparagine synthetase has only been recently identified, following the characterization of four families presenting with congenital microcephaly, intellectual disability, progressive cerebral atrophy, and intractable seizures [197]. Probands display reduced levels of asparagine synthetase with suggested loss of function and the human symptoms were mimicked in mice modified to express the gene at lower levels although the mice showed no epileptic activity. The authors proposed that these neurological impairments could result from either asparagine depletion in the brain or by accumulation of aspartate/glutamate, leading to enhanced excitability and neuronal damage. Aspartate and glutamate were mildly elevated in both urine and plasma of affected individuals.

**Fig. 6:**
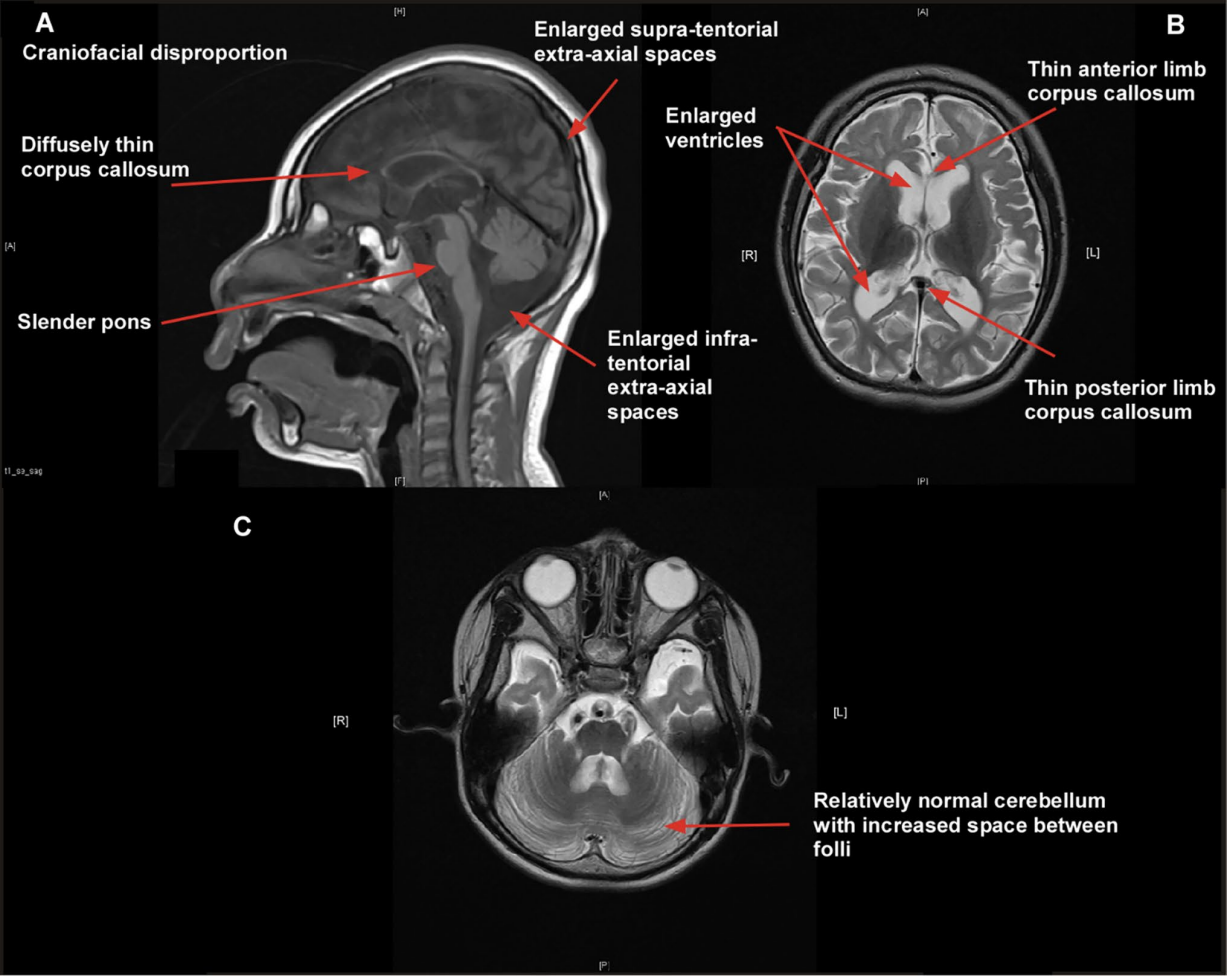
MRI of 4 year old boy with compound heterozygous pathogenic variants in *ASNS* (NM_183356.3:c. [866G > C]; [1010C > T]). **A** sagittal and **B**, **C** axial T2-weighted images showing small cranial vault with craniofacial dysmorphism in keeping with microcephaly, enlargement of the ventricles and the supratentorial and infratentorial extra-axial spaces in keeping with global atrophy in both the supratentorial and infratentorial compartments. Also noted are a diffusely thin corpus callosum, slender brainstem and a generalized, marked reduction of white matter volume. Head circumference was between − 1 and − 2 SD from the mean at birth but rapidly decelerated to be below − 5 SD from the mean by 20 months of age [200]

Asparagine synthetase is expressed strongly in the cortical plate in developing embryonic mouse brain and also in the ventricular and subventricular zones where neuronal progenitor cells are generated [198, 199] suggesting that asparagine is required for neuronal development. Recently, fibroblasts cultured in low asparagine media have shown markedly reduced proliferation, providing evidence for the importance of asparagine in cell growth [200]. These authors reconfirmed the key neurological finding of the disorder as markedly progressive cerebral atrophy (Fig. 6).

The epileptic activity seen in asparagine synthetase deficiency is currently thought to result from elevated glutamate [201], a substrate for asparagine synthesis (Fig. 5) along with aspartate. A recent attempt to treat a patient with asparagine synthetase deficiency with supplemental asparagine resulted in a temporary improvement of symptoms, with a subsequent worsening of seizures, sleep disturbance, and increased irritability [202]. However, accurate attribution of symptoms to alterations in asparagine metabolism or to one or other of the other reactants of the enzyme will require further investigation [196].

### Synthesis of Purine Nucleotides

l-Aspartate is a substrate for the de novo synthesis of purine nucleotides as well as in the recycling of purines (Fig. 5). Although some limited ability to synthesize purines de novo has been reported in brain, the exact pathways are not well described and the capacity has been reported to vary with developmental stage [203, 204]. l-Aspartate is a substrate for adenylosuccinate synthase (ADSS), which generates *N*−6-(12-dicarboxyethyl)AMP (Adenylosuccinate; S-AMP) from inosine monophosphate and l-Aspartate, with the carbon backbone being released in the subsequent reaction as fumarate. Incorporation of the nitrogen from aspartate has been shown to be concentration dependent in astrocyte cultures, with formation of [6-amino-^15^N]nucleotides occurring when incubated with 2.5 mM [^15^N]aspartate but to a much less degree at 0.5 mM [^15^N]aspartate [177].

#### Defects in Adenylosuccinate Synthase

Described deficiencies in the *ADSS* gene are rare, indicating a conserved role for the enzyme. A series of 13 patients has been described with submicroscopic pure deletion of distal 1q with common features including severe mental retardation with serious speech impairment, hypotonia and a motor developmental delay and corpus callosum abnormalities with seizures present in more than half of the group. Four candidate genes were isolated including *ADSS* [205].

### Aspartyl-tRNA; Synthesis and Defects

Aspartate is also a substrate for aspartyl-tRNA synthetases (DARS; aspartyl-aminoacyl-RNA-synthetase), whose role is to accurately pair the appropriate t-RNA with aspartate and ensure exactitude in protein synthesis (Fig. 5). There are both mitochondrial and cytosolic forms of these enzymes (DARS2 and DARS, respectively). A recent study has identified the expression patterns of human DARS mRNA and DARS protein in healthy brain [206]. mRNA expression mirrors that of protein closely with the highest level of expression in the cerebellum at around 50% higher than the other regions examined, which included cortex, brainstem, hippocampus, striatum and corpus callosum. Expression patterns of the mitochondrial *DARS2* were similar to that of *DARS*. Across these brain regions, the protein was expressed in oligodendrocytes, astrocytes and microglia but expression was consistently much stronger in neurons [206].

Mutation in *DARS2* causes Leukoencephalopathy with Brain stem and Spinal cord involvement and elevated Lactate (LBSL; [207]) while homozygous and compound heterozygous point mutations of the *DARS* gene cause the white matter disorder Hypomyelination with Brain stem and Spinal cord involvement and Leg spasticity (HBSL; [208]). Similarities between these two disorders suggest the diseases may share a common underlying pathology, though the exact mechanism has not yet been identified [209]. Complete disruption of the *Dars* [209] or *Dars2* [210] gene in transgenic mice is embryonic lethal suggesting that a lack of enzymatic activity of one enzyme cannot be compensated by action of the other.

Patients with a late (adolescent) onset disease caused by *DARS* mutations have been described as presenting with a condition that mimics an acquired, steroid-responsive, inflammatory disease such as multiple sclerosis [211]. Diffuse T2 white matter hyperintensity is a feature of the disease with almost all supratentorial white matter regions affected, as well as superior and inferior cerebellar peduncles, the anterior brainstem and sections of the spinal cord.

It has been noted that the enzyme aspartyl-tRNA synthetase, differently from all other amino acyl-tRNAases, does not distinguish between D- and l-Aspartate [212] meaning that d-aspartate can be incorporated into proteins. This could have practical implications for studies of age-related neurodegenerative diseases since it has been shown that the content of d-aspartate in protein lysates prepared from human brain tissue, in a manner preserving the chirality of amino acids, increased with age and was significantly higher in persons with Alzheimer’s disease[213]). How could such differences come about?

It has been suggested that the homochirality (L-configuration) of amino acids in proteins decreases with age (as the overall entropy of the organism increases with time [214], and in theory, this could contribute to the higher content of d-aspartate in brain proteins of older individuals (and those with Alzheimer’s disease in particular). However, the rate of spontaneous racemization of proteins in vivo would be difficult to establish with any precision because of the rather rapid turnover of most proteins (half-lives in hours to days; [215]), probably exceeding the rate of racemisation by orders of magnitude; in proteins with slower turnover (connective tissue) the racemization is, at least for aspartate, of the order of 1% per year (review: [214]). This makes the spontaneous change of configuration a very improbable mechanism of the “natural” racemization of d-aspartate in proteins, whether they come from healthy or Alzheimer’s disease brains. The answer must be sought in a differential availability of d-aspartate for protein synthesis under such (pathological) conditions.

Production of free d-aspartate (by racemization) in brain tissue is catalysed by aspartate racemase (EC 5.1.1.13; see also Sect. “The Presence and Functions of d-aspartate in the brain.” further in the text), leading to a high content of d-aspartate in brain, at least early in ontogeny. d-aspartate is then gradually removed as the expression and activity of a d-aspartate converting enzyme d-aspartate oxidase (DDO, EC 1.4.3.1) increase (review [216]). DDO interacts with pLG72, a human-specific protein residing in mitochondria and this may not only regulate the degradation of DDO but also restrain the generation of radical oxygen species (RSO) by the DDO reaction (reviews: [216–218]). As the activity of DDO increases later in development (by gradual demethylation of *DDO* at 6q.21), the content of d-aspartate in brain becomes low and remains low throughout adulthood (review: [216]). In senescent brains and, in AD in particular, the fine regulation of d-aspartate levels (and the concomitant production of RSO) in brain could be altered, perhaps by changes in posttranslational modification of DDO protein and methylation of the DNA (*DDO* at 6q.21 [219]; reviews: [214, 220]), or, analogous changes in pLG72 (or the gene *G72* 13q33.2; [221]). This could explain, why the levels of free d-aspartate in Alzheimer’s disease increase and why more d-aspartate might end up in proteins [213].

## Transport of Aspartate

Both d- and l-aspartate are avidly transported by Na^+^-dependent “high affinity” glutamate/aspartate transporters (excitatory amino acid transporters, EAAT1-5) encoded by five genes (EAAT1/GLAST, *SLC1A3*; EAAT2/GLT, *SLC1A2*; EAAT3/EAAC1, *SLC1A1*;EAAT4, *SLC1A6*; EAAT5, *SLC1A7* [222, 223]) highly expressed in brain in the form of several splice variants [224, 225]. These are usually discussed in the context of synaptic signalling mechanisms (cf. the next section). Altered function of EAATs has been implicated in several disorders (see examples in Table 2) but since these transporters play a major role in the uptake of the excitatory neurotransmitter l-glutamate, it is difficult to ascribe disease mechanisms to either aspartate or glutamate, with glutamate implication the more probable [226].

**Table 2:** Neurological and mental disorders potentially linked to disturbed function of excitatory amino acid transporters (EAATs)

Transporter	Disorder	References
EAAT1/GLAST	Alcoholism	[227–230]
	Alzheimer’s disease	[231]
EAAT2/GLT1	Manganese toxicity	[232, 233]
	Amyotrophic lateral sclerosis	[234–236]
	Schizophrenia	[237–239]
	Alzheimer’s disease	[240]
	Ischaemia/hypoxia	[241]
EAAT3/EAAC1	Alzheimer disease	[242]
EAAT5	Macular degeneration	[243]

A sodium-dependent transporter of glutamine that is inhibited by d-aspartate has been described in cultured neurons [244] but its molecular nature remains uncertain. It is apparent that aspartate can efflux from brain cells, particularly from astrocytes during conditions such as hypoglycaemia [65] but the transporter that likely mediates this is unknown.

l-Aspartate interacts only very poorly with the “low affinity” Na^+^-independent glutamate/cystine exchanger in C6 glioma cells and astrocytes [245]. Both enantiomers of aspartate were only moderate inhibitors (at mM concentrations) of Na + -independent l-cystine uptake in brain but this may be explained by a very weak interference with a histidine-selective transport system which might have been taking up l-cystine [246]. It is not known whether these low affinity transporters have any specific significance for aspartate traffic in brain tissue. A transporter (ASCT2; SLC1A5) that handles l- but not d-aspartate, has been identified in cultured mouse and rat brain endothelial cells used as a blood brain barrier model in vitro [247]. This transporter could be responsible for the stereospecificity of aspartate efflux reported earlier at the BBB [247, 248] although other authors report that ASCT2 has a very low affinity (K_M_ > 60 mM) for l-aspartate [249] and that l-Aspartate becomes a relevant substrate only at low pH (5.6 [250]). ASCT2 is expressed at high enough levels to be studied in some detail in the BBB model endothelial cells [247] and its expression in the mouse brain, as shown by immunohistochemistry, seems indeed restricted to the abluminal side of brain capillaries, thus implying a possible role at BBB [247] A protein (sialin; reviews [251, 252]) was proposed to act as the aspartate transporter in presynaptic vesicles but see also [253] and the next section for further discussion and criticism.

## l-Aspartate as a Signalling Molecule in the Brain

### Exogenous Aspartate Changes Brain Activity?

The story of excitatory amino acids glutamate and aspartate started in the 1940’s in Japan when Takashi Hayashi applied glutamate and aspartate to the cerebral cortex of dogs and noted that it produced increased motor activity and convulsions. Some of the findings were published in Japanese before the end of World War II [254] but reports in English only appeared in the 1950 s ([255]; see [256] for a full historical review). Testing aspartate was a part of these experiments [256]..

### Aspartate as an Excitant of Central Neurons

In the late 1950 s and early 1960 s Curtis and Watkins tested a number of amino acids, including l-glutamate and l-Aspartate on single cells of the cat spinal cord [257–259]. These studies were crucial in establishing both l-glutamate and l-Aspartate as the leading candidates for the role of excitatory synaptic transmitters (“neurotransmitters”) in the central nervous system (CNS; for a historical review see [260]).

Graham et al. [261] were among the first to suggest that the roles of glutamate and aspartate as neurotransmitters could vary from one CNS region to another, at particularly within the spinal grey matter. Together with a follow-up lesion study [262] these findings were, interpreted as aspartate mediating the signal transmission at the synapses of the local excitatory interneurons while glutamate is released by the axon terminals of the primary sensory afferents. Duggan [263] claimed to have found differences between actions of glutamate and aspartate at interneurons and Renshaw cells in the ventral horn thus, perhaps, further refining the hypothesis. In general, however, the idea that aspartate acts as a neurotransmitter “in its own right”, received only sporadic attention and unconvincing or conflicting evidential support ([264, 265]; for reviews and additional historical notes cf. [266]). This does not mean that aspartate ceased to be thought of as a putative neurotransmitter; it continued to be considered as a co-transmitter presumably released with glutamate, for example in the central visual pathways [267–270] or in the hippocampus [271] and elsewhere [272, 273]. In time, however, the case of neurotransmitter aspartate was getting more complicated.

### The Intriguing Case of Neurotransmitter Aspartate

In order to be considered a neurotransmitter, a compound has to conform to a set of criteria [274](reviews: [271, 275]; historical notes: [260]) as follows:

1. synthesis and storage at the synapse;
2. stimulus-coupled Ca^2+^-dependent release from the presynaptic nerve terminals;
3. physiological and pharmacological identity of action with the endogenous neurotransmitter and,
4. the presence of a specific and efficient inactivating mechanism located either in the nerve terminals or in adjacent structures e.g. astrocytes.

One could add metabolism and recycling but, given that both glutamate and aspartate avidly participate in a variety of reactions and biochemical pathways in almost any cell and tissue, such criterion would seem to be of little practical import in this case.

Starting with criterion 4, a very efficient Na^+^-dependent transport system for l-glutamate was soon identified [276]. From the time the transport was first described (synaptosomes: [277, 278]; brain slices: [279]; reviews: [223, 280]) both l-glutamate and l-aspartate were studied as substrates in parallel experiments. Significantly, this was also the case in the early studies using cultured glial cells ([281]; reviews: [282, 283]). While the existence of a weak transport system preferring glutamate over aspartate and associated with neuronal structures could not be ruled out ([284]; for further discussion and additional references see [283, 285]), the most potent and abundant glutamate/aspartate transporters were eventually found to reside mostly in astrocytes (reviews: [222, 223, 286, 287]). In the meantime, radiolabelled [^3^H]d-aspartate emerged as a marker of choice in studies of the Na^+^-dependent glutamate/aspartate transport, [288] having—as it was widely assumed—an advantage over [^3^H]l-glutamate and [^3^H]l-aspartate in not being rapidly metabolized (e.g. [289–291]; reviews: [283, 292]). In experiments using frozen brain sections and performed at temperatures near 0 °C one could also employ [^3^H]l-Aspartate [293, 294]. This approach does not test the transport mechanism itself, rather it looks at a binding site which displays all main characteristics of the glutamate/aspartate binding site on glutamate/aspartate transporters and enables accurate determination of binding affinities of principal ligands. These studies resulted in a characterization and quantification of interesting heterogeneities in the nature of the Na^+^-dependent glutamate/aspartate binding sites some of which had been detected earlier in transport and binding studies ([295–298]; for a review and analysis of relevant data see [299]) and indicated that there could be an l-aspartate-preferring form of the transporter that appeared to be particularly strongly expressed in the cerebellar cortex ([300], review: [292]).

In theory, the regional variations in [^3^H]l-Aspartate binding could be explained by the presence of as yet unidentified high affinity glutamate/aspartate-selective binding sites in brain tissue, or, more specifically, by the existence of an unknown protein molecule with characteristics similar to those of the other five EAATs. A more parsimonious (and perhaps more plausible) explanation would focus on possible regional variations in the properties and distributions of the numerous splice variants of existing EAATs [224, 237] which continue to be uncovered [301, 302]). Characteristics of glutamate/aspartate binding sites on EAATs such as the affinity, substrate specificity and ionic dependence are quite restrictive [279, 303]; reviews: [223, 292, 304] and would clearly distinguish the EAAT-located binding sites from those on the excitatory amino acid receptors (reviews [223, 292]) or related enzymes (e.g. glutamate decarboxylase [305, 306]:). There are occasional exceptions such as the interaction of l-serine-*O*-sulphate with glutamate transport [279], [^3^H]l-Aspartate binding [300] and EAAT function [307] on one hand and aspartate aminotransferase (AAT) on the other; the criterion of Na^+^-dependence would, however, adequately distinguish a transporter binding aspartate on the outside of the plasma membrane from the AAT active in the cytoplasm. The possible existence in brain of an aspartate-preferring variant of EAAT (or one of the related family of ASCT; [308]) should, therefore, be considered; in the context of l-Aspartate (and d-aspartate, see below) being investigated as putative neurotransmitters, it could be significant.

It took somewhat longer to establish the identity of action (criterion 3). This was probably because the receptors at which glutamate was supposed to act turned out to be much more diverse and complex than originally thought ([309]; historical overview: [266]) and an adequate range of specific antagonists, therefore, took time and effort to find (reviews: [303, 309]). The matter was further compounded by the discovery of the metabotropic glutamate receptors (mGluRs; linked to G-proteins; review: [310]. Some studies indicated that aspartate was about equal to glutamate as an activator of the metabolic reactions linked to mGluRs [311]while in other cases, it appeared inactive or even as a weak antagonist [312, 313]. As a good substrate for the Na^+^-dependent glutamate/aspartate transport, aspartate could, of course, activate mGluR indirectly, via glutamate “washed out” of the cells by the transport-mediated heteroexchange [314]. It seems that aspartate has never been systematically tested at mGluR’s and may well be near-inactive there as a ligand, except, perhaps, at atypical mGluR’s such as those reported by Kanumilli et al. [315] to exist in glial cells. The nature of such receptors is unknown, but instances of functionally—but not necessarily structurally—identified novel mGluR’s do emerge in the literature occasionally [316, 317], see [318] for a review); whether such mGluR are aspartate sensitive or not has not been established, though. Alternatively, the observed aspartate sensitivity of mGluR receptors [315] may have resulted from an interplay between NMDA-receptors (which are aspartate-sensitive) and metabotropic receptors (review: [319]); the issue remains unresolved.

As all ionotropic glutamate receptors were activated by aspartate, the status of aspartate as a possible (glutamate co-) transmitter would not be threatened by the discovery of apparently aspartate insensitive mGluRs. Nor did the demonstration of glutamate and aspartate release (criterion 2) from stimulated preparations such as synaptosomes, posed, at least initially, any apparent challenge [320]. The really serious problem for advocates of the neurotransmitter role of aspartate was encountered when the studies of the neurotransmitter release were taken a step further (upstream) and the accumulation and storage of the excitatory amino acids in synaptic vesicles (criterion 1) began to be investigated.

For a neurotransmitter to be effectively sequestered and stored in synaptic vesicles, it needs to be accumulated by a specific active transport, usually driven by electrical and/or H^+^-gradient (or H^+^-dependent ATPase; [252, 321]). In the case of l-glutamate such transport was studied using preparations enriched in synaptic vesicles [322, 323]and while the transport system readily accumulated both l- and D-glutamate, it showed little or no affinity for aspartate. Did this mean that previously reported stimulated release of aspartate from brain tissue (e.g. [273, 324, 325]) was not a product of synaptic release? The apparent lack of an aspartate-accumulating uptake system in synaptic vesicles would have created particular conundrum for researchers (e.g. [298, 326]) who investigated the synaptic release of glutamate using, as an experimental model, preparations preloaded with the radiolabelled “non-metabolizable” analog d-aspartate [288]. If the synaptic vesicles did not take up and, therefore, presumably could not store, d-aspartate, where did the radiolabel (^3^H or ^14^C) come from in those studies? Could it be accumulated and released by a glial-located compartment [327]? If so, how would we explain the depolarization-coupled Ca^2+^-dependent release of endogenous aspartate from highly purified synaptosomal fractions ([328] review [329]) and other similar observations?

Cloning and characterization of vesicular transporters took a long time to come and, when the results finally appeared (for reviews see [252, 321, 330]), they were of little help to those who still hoped to show that aspartate was a neurotransmitter. As predicted by the earlier uptake studies, all three cloned vesicular glutamate transporters (vGLUT1, *SLC17A7*; vGLUT2, *SLC17A6*; vGLUT3, *SLC17A5*) handled glutamate but none accepted aspartate [330]. More recently, however, a potential vesicular transporter that handles aspartate (vesicular excitatory amino acid transporter, VEAT or *SLC17A5)* has been cloned ([251]; reviews: [252, 271, 321]). As with vGLUT1–3, VEAT is a multifunctional protein (sialin, the “primary” function seems to be to transport sialic acid out of the lysosomes; [251]) but it remains to be shown if it is indeed located in synaptic vesicles and/or significantly contributes to the transport of aspartate across their membrane [331]. As of early 2025, the evidence, albeit tenuous, continues to imply that the transporter might exist but has not yet been identified [253, 332, 333].

The above controversies re synaptic accumulation and Ca^2+^ dependent, stimulus-coupled release have somewhat eroded the proposed status of l-Aspartate as a neurotransmitter; clearly more research needs to be done.

## The Presence and Functions of d-aspartate in the brain

The presence of D-amino acids and their metabolizing enzymes in the mammalian tissues has been known for decades [334] and in 1969 de Marchi and Johnston noticed that sheep brain contained an enzymic activity that readily oxidized glycine and several D-amino acids, including d-aspartate. Moreover, using cat spinal cord tissue, they obtained evidence that the enzyme activity was particularly high in grey matter [335]. In a subsequent study, Davies and Johnston [288] used extracts of sheep brain to demonstrate the presence of a specific d-aspartate oxidase (DDO) in the central nervous tissue that showed similarities to the analogous enzyme activity earlier detected in kidney (cf. also [336]; for a review, additional references and more detailed discussion see [337]). The idea that d-aspartate is present in central nervous tissue and has (a) specific role(s) in brain function was in line with the discovery that the “high affinity” Na^+^-dependent glutamate transport, while strongly preferring L- over D-glutamate, handled both D- and l-Aspartate about equally well ([279, 288]; this was subsequently termed a “stereoselective anomaly” of glutamate transport systems (review: [338]; notes on the mechanism [339]).

As already indicated above, d-aspartate was initially regarded merely as a convenient “non-metabolizable” marker in studies of glutamate transport either as a radiolabelled ligand and/or tracer ([288, 290, 291]; reviews: [223, 280, 283, 340]) or as a non-radioactive ligand fixed by glutaraldehyde in situ and subsequently detected by immunohistochemistry ([341–344]). In the meantime Dunlop et al. [345] reported high levels of endogenous d-aspartate in the mammalian central nervous tissue, particularly during development (reviews: [346, 347]), leading to questions regarding possible role(s) of d-aspartate in brain [348].

Most of the subsequent work on d-aspartate in brain tissue came from a few laboratories mainly in the US and Italy ([349–356]; reviews: [357–359]) and provided indications that d-aspartate could have specific functions in brain. There is little evidence that d-aspartate acts as a neurotransmitter, (as has been advocated by some [350, 360]), but its chief importance in the brain seems to rest mainly with its regulatory roles in neuroendocrine functions and glutamate homeostasis. As in the case of l-Aspartate, the status of d-aspartate as a neurotransmitter, therefore, appears at best “on hold” [288, 361].

Evidence that d-aspartate plays a part in the function of mammalian brain comes from a range of biochemical, histological and behavioural experiments (for a summary and review see [362]). In essence, d-aspartate is prominently present in embryonic brain but the levels tend to decrease postnatally. This apparently results from a rapid postnatal increase in the activity of d-aspartate oxidase which is the principal d-aspartate metabolizing enzyme in brain [363]. As d-aspartate acts on NMDA receptors [364, 365] and aberrant development and/or deficient activity of NMDA receptors has been linked to the etiology of schizophrenia ([366]; reviews [367, 368]), one may ask whether altered levels of d-aspartate might not also be involved in the process. Errico et al. [369] demonstrated that experimentally increased levels of d-aspartate—achieved either by d-aspartate administration or by DDO knockout—attenuated characteristic behavioural responses in animal models of schizophrenia (induced either by amphetamine or by the NMDA receptor blocker MK801/dizocilpine). d-aspartate even appeared to mimic the effects of the neuroleptic haloperidol [369]. In humans, it has been shown in patients with the disease that the dorsolateral prefrontal cortex (one of the main regions of interest in schizophrenia [370]) displayed higher activity of DDO and lower levels of d-aspartate [371]. Recent findings of lower d-aspartate levels in blood sera of patients with schizophrenia [372] raised hopes that measuring d-aspartate serum levels could be employed as a diagnostic tool [373].

Moreover, it is not just d-aspartate metabolism what might be altered in psychosis; the d-aspartate metabolite NMDA (Fig. 5, 5.10) levels (in addition to those of D- but not l-Aspartate) have also been reported as about 60% lower in the prefrontal cortex of SCZ patients [374]. NMDA appears to be normally present in brains of humans at levels about an order of magnitude lower than those of d-aspartate [374], an NMDA-synthesising enzyme activity (d-aspartate *N*-methyltransferase; DDNMT, EC 2.1.1.XX) seems to exist in various mammalian tissues including the rodent brain [375] and Na^+^-dependent NMDA uptake and release (K^+^-stimulated, Ca^2+^-independent), both studied using radiolabelled *N*-[methyl-^3^H]-d-aspartic acid), were also demonstrated in rat brain slices in vitro [376].

In this context, it might be interesting to note that perturbed expression of GLT1 (EAAT2, *SLC1A2*), the principal transporter mediating about 95% of the transport and clearance of l-glutamate (and potentially l- and d-aspartate) from the extracellular space in brain [286] could also be involved in the hypothetical d-aspartate dysfunction in schizophrenia. Some of the initial evidence came from animal studies showing that GLT1/EAAT2 was downregulated by 70% in brains of rats given the neuroleptic clozapine [377], see also [239] for a review and more references) thus indicating that antipsychotics such as clozapine might, at least in part, act by decreasing the clearance and increasing the extracellular levels of acidic amino acids including d-aspartate. It was subsequently shown that brains of non-medicated patients with schizophrenia displayed higher expression of EAAT2, particularly in the prefrontal cortex [238]. It was interpreted in terms of the glutamatergic hypothesis in schizophrenia (review: [239]) but would apply to and be equally well consistent with, the proposed d-aspartate deficiency.

Another line of evidence comes from experiments linking d-aspartate to the levels of steroid (sex) hormones in the brain. It has been shown that administration of d-aspartate, both chronic oral and acute intraperitoneal, to adult rats, dramatically increases the brain levels of progesterone, testosterone and 17β-estradiol [378]. This could be of significance given that steroid hormones have been known to be neurotrophic and can stimulate both synaptogenesis and neuroregeneration [379–381]; particularly so, if the hormones were under the control of d-aspartate. Recent evidence indeed suggests that d-aspartate can increase dendritic spine density in rat hippocampal slices and that this can translate, when large doses of d-aspartate are given to rats *per os*, to increased functional connectivity in hippocampus [382]. Whether the above observations indicate the existence of a mechanism involving d-aspartate (upstream of the sex hormones) and regulating processes such as synaptogenesis and/or neuroregeneration even in the adult brain is too early to decide. The potential value of further research in the area seems beyond dispute.

The above findings are indicative of the important role of d-aspartate in brain function particularly in relation to an increased risk of psychosis [359]. Higher levels of d-aspartate may not be always beneficial [383] and much more will have to be known of the d-aspartate-containing structures in brain; current evidence suggests that endogenous d-aspartate exists in neuronal perikarya in many parts of the central nervous systems including the cerebellum but not the cerebral cortex [384].

## Measurement of Aspartate in the Brain with Magnetic Resonance Spectroscopy

Aspartate contributes two coupled sets of resonances to the ^1^H magnetic resonance spectrum from the αCH and βCH_2_ protons, forming a typical second order “ABX” spin system yielding four (doublet of doublets, 3.89 ppm) and eight (a pair of doublet of doublets; 2.65 and 2.80 ppm) resonance lines, respectively [385]. Many of the CH_2_ resonances are obscured by the equivalent resonances from NAA but the higher frequency components of the CH_2_ spin system can be resolved at 3 T, allowing some relatively unadulterated, albeit suboptimal, input for fitting routines. The αCH proton at 3.89 ppm is well resolved from the equivalent NAA αCH proton, which is found at 4.38 ppm.

Aspartate measurements by magnetic resonance spectroscopy (MRS) have not been reported frequently due to the inherent difficulties in measuring an amino acid present in low concentration and with relatively low signal to noise due to resonance splitting and overcrowding in the frequency domain. The incentive for going to the trouble to measure aspartate is compromised by the lack of knowledge of any clear role for it, while measurement of it by MRS may assist with our understanding of what its role(s) in the brain consists of. With improvements in magnetic resonance technology, including the introduction of spectral editing approaches for aspartate, the molecule is now starting to make an appearance in the MRS literature.

Standard one-dimensional spectral localization techniques such as PRESS (Point Resolved SpectroScopy; [386, 387]) and STEAM (Stimulated Echo Acquisition Mode; [388]) can be used to measure aspartate but judicious choice of echo time is needed. Reliable and reproducible results have been obtained at 7 T using the s-LASER (semi-LASER, Localisaton by Adiabatic SElective Refocussing) sequence [13] which gives less chemical shift displacement error than PRESS and allows for shorter echo times [389].

The J-resolved PRESS (J-PRESS) has been reported to be a less reliable method for measuring aspartate [390] as has CEST (Chemical Exchange Saturation Transfer), either because of low levels of aspartate in brain, poor exchange of its protons with water or unfavorable exchange rates in vivo [391].

**Fig. 7:**
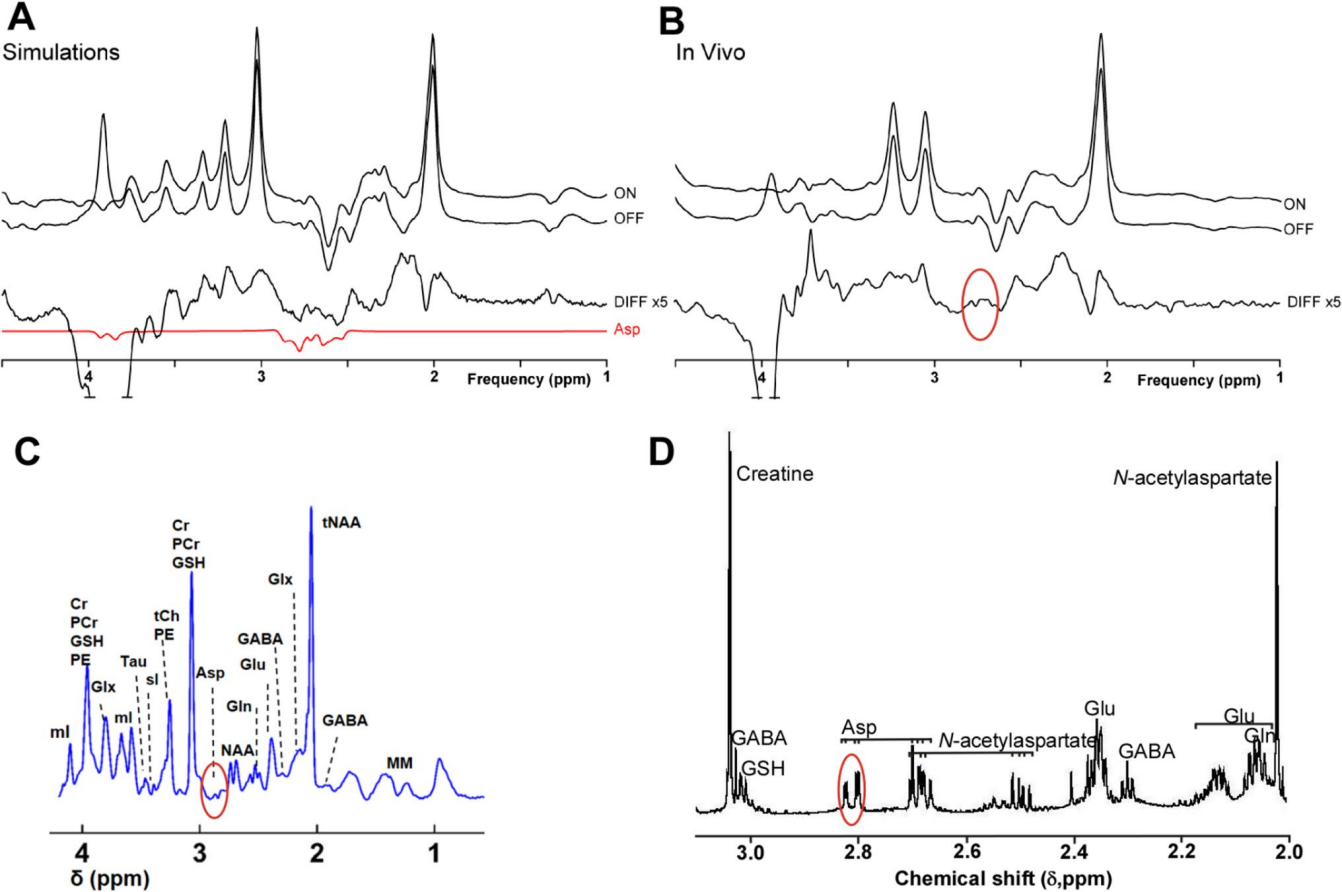
^1^H Spectra of brain showing aspartate resonances. Panels A and B show simulated (**A**) and actual (**B**) in vivo MEGA-PRESS (TE = 150 ms, ON frequency 3.89 ppm, OFF 10.0 ppm, editing pulse 45 ms, 192 averages, VOI 27 cm^3^ in right centrum semiovale, 32 channel head coil) spectra of human brain at 3 Tesla. The spectrum of edited aspartate is shown in red (**A**). **C** shows a section of a spectrum of human brain in vivo acquired at 9.4 T with a home-built multi-transmit-receive coil using a metabolite-cycled semi-LASER sequence (TE = 24 ms, VOI 8 cm^3^ in occipital lobe, 96 averages) [22]. mI, myoinositol; Cr, creatine; PCr, phosphocreatine; GSH, glutathione; PE phosphoethanolamine; Glx, glutamate and glutamine; Tau, taurine; tCh, total choline; tNAA, *N*-acetylaspartate and *N*-acetylaspartylglutamate; MM, macromolecules. **D** shows a section of a high field NMR spectrum of Guinea pig cortex extracted with methanol/chloroform [396] acquired at 800 MHz (18.8 T). The high frequency resonances from the aspartate CH_2_ protons are shown inside red ellipses

It is possible to selectively edit spectra for aspartate, for example using the MEGA-PRESS sequence [392] which uses selective irradiation of coupled resonances combined with acquisition of an unedited spectrum to produce an edited spectrum following subtraction of the edited from the unedited spectrum. The approach has been described for editing aspartate using an echo time (TE) of 80 ms [393], with 115 ms TE [394] and also editing aspartate simultaneously with NAA and NAAG, the so-called HERMES approach [18] using an echo time of 150 ms in which the edited aspartate CH_2_ resonances show maximum inversion. Even using this approach, the signal we seek is of low intensity compared to the noise and is dispersed due to J-coupling and the relatively long echo time required for better resonance refocusing. An example of a MEGA-PRESS editing approach at 3 T is shown in Fig. 7, with subtraction of a selectively edited spectrum from an unedited spectrum yields resonances that can be fitted with a simulated or acquired basis set.

The better frequency range available at 9.4 T (400 MHz) allows resolution of some of the aspartate βCH_2_ protons from those of NAA [22] but the signal is, by nature of its low concentration and ABX coupling pattern, of low signal to noise (Fig. 7C) and it is not fully resolved from the aspartyl moieties of NAA, even at field strengths as high as 18.8 Tesla (Fig. 7D. Use of an ultra-short TE at high field (9.4Tand 10.5 T with FID-MRSI)[395] has allowed single slice [397] or whole brain mapping of aspartate [398] which indicates that aspartate distribution tends to mirror glutamate more than that of *N*-acetylaspartate.

In summary, precise measurement of aspartate levels in human brain in vivo currently remains the preserve of those dedicated to careful spectroscopy.

## Future Directions

Aspartate is a key molecule in brain metabolism, playing multiple interdependent roles and acting as a pivotal metabolite in diverse pathways. There remains much to learn about this enigmatic molecule including details of its transport and compartmentation, whether or not it really is a neurotransmitter and under what circumstances, its putative role in seizure generation, how it interacts with the brain metabolite *N*-acetylaspartate and how levels of aspartate relate to malate-aspartate shuttle activity to name just a few aspects that remain poorly understood.

Aspartate remains absent from most models of brain metabolism. It is unclear what difference it would make to the outcomes were it to be included, particularly given the necessarily reductive nature of many of these models and the rapid exchange kinetics of the reaction catalysed by aspartate transferase although its role in the transfer of reducing equivalents via the malate aspartate shuttle suggests that models of short term (seconds) brain activity may benefit from its incorporation. Improvements in the measurement of it, in vivo, and increased frequency of doing this will aid in our understanding.

## Data Availability

No datasets were generated or analysed during the current study.
